# Maternal Diet During Pregnancy and Blood Cadmium Concentrations in an Observational Cohort of British Women

**DOI:** 10.3390/nu12040904

**Published:** 2020-03-26

**Authors:** Caroline M. Taylor, Rita Doerner, Kate Northstone, Katarzyna Kordas

**Affiliations:** 1 Centre for Academic Child Health, Bristol Medical School, University of Bristol, 1–5 Whiteladies Road, Bristol BS8 2NU, UK; 2 Population Health Sciences, Bristol Medical School, University of Bristol, Oakfield Grove, Bristol BS8 2BN, UK; rita.doerner@charite.de (R.D.); kate.northstone@bristol.ac.uk (K.N.); 3 Department of Epidemiology and Environmental Health, School of Public Health and Health Professions, University at Buffalo, Buffalo, NY 14214, USA; kkordas@buffalo.edu

**Keywords:** ALSPAC, diet, dietary patterns, blood cadmium, pregnancy

## Abstract

Few studies have investigated the extent to which diet predicts body Cd concentrations among women of reproductive age, and pregnant women in particular. The aim of this study was to examine diet as a predictor of blood Cd concentrations in pregnant women participating in the UK Avon Longitudinal Study of Parents and Children (ALSPAC). Whole blood samples were analysed for Cd (median 0.26 (IQR 0.14–0.54) µg/L). Dietary pattern scores were derived from principal components analysis of data from a food frequency questionnaire. Associations between dietary pattern scores and foods/food groups with blood Cd ≥ median value were identified using adjusted logistic regression (*n* = 2169 complete cases). A *health conscious* dietary pattern was associated with a reduced likelihood of B-Cd ≥0.26 µg/l (OR 0.56 (95% CI 0.39–0.81)). There were similarly reduced likelihoods for *all leafy green and green vegetables* (0.72 (0.56–0.92) when consumed ≥4 times/week vs ≤1 to ≥3 times/week) and with *all meats* (0.66 (0.46–0.95) when consumed ≥4 times/week vs ≤ once in 2 weeks). Sensitivity analysis excluding smokers showed similar results. The evidence from this study provides continued support for a healthy and varied diet in pregnancy, incorporating foods from all food groups in accordance with national recommendations, without the need for specific guidance.

## 1. Introduction

Metal contaminants may be incorporated into edible plants during cultivation or settle on plant surfaces at any time during food production, transport, or processing, thus contributing to human exposure [1,2]. Food is the main source of cadmium (Cd) exposure in non-smoking populations [3], and this is particularly concerning in vulnerable groups, such as pregnant women. There are no known safety thresholds for metal exposure and no recommendations for acceptable blood or urine Cd concentrations specifically during pregnancy [4]. Estimated intestinal absorption ranges from 1% to 11% [5] but are thought to increase during pregnancy, at least in laboratory animals [6]. Cd accumulates in the placenta [7] and is detectable in cord blood and plasma, albeit at lower concentrations than in the pregnant woman’s blood [8,9,10]. Nevertheless, maternal Cd exposure has been linked with adverse pregnancy outcomes, including lower birth weight [7,11,12].

The World Health Organization [13] lists Cd among its priority contaminants for monitoring in total diet studies. Across several studies, risk of exposure from dietary sources has been found to be generally low, but some foods, such as vegetables (particularly mushrooms), rice, seafood (particularly bivalves), products made with cacao beans, and offal, have particularly high Cd concentrations [14,15,16,17,18,19,20], although foods that are more frequently eaten are likely to make the greatest overall contribution to dietary Cd exposure [21]. There is some evidence that food consumption patterns may influence the contribution of the diet to daily Cd exposure. For example, among 50–79-year-old women participating in the US Women’s Health Initiative, higher consumption of vegetables and grains was associated with higher dietary Cd exposure [22]. Among pregnant women from Mexico, the urinary Cd concentration measured during the third trimester was positively associated with estimated Cd in the diet but only among non-smokers [23]. A further study, however, found no associations between a range of food items and blood Cd concentrations (B-Cd) in premenopausal women in the USA [24].

Relative absorption of Cd can be affected by a number of factors. Cd typically occurs as a divalent element and can be absorbed via several intestinal transporters. Chief among these is the divalent metal transporter-1 (DMT1), typically responsible for the absorption of iron [18,25]. In pregnant rats, the upregulation of DMT1 in the latter part of pregnancy is the most likely explanation for higher Cd absorption and at least twofold higher Cd accumulation in the intestine, liver, and kidney compared with non-pregnant controls [6]. Low iron status, which induces DMT1 expression, has been associated with higher blood Cd concentrations in both pregnant and non-pregnant women of reproductive age who were non-smokers [26,27,28]. In smokers, concentrations of serum ferritin (a biomarker of iron stores) were inversely associated with B-Cd only among women who were relatively light smokers (<5 cigarettes/day) [29].

Cd in plant foods, however, occurs in complexes with nutrients or bioactive components, which may also affect the absorption of Cd from the diet [30]. For example, divalent metals may counteract the absorption of Cd in the intestinal tract or promote the excretion of Cd [31]. Among US adults aged 20 years or older, total dietary zinc intake (from diet and supplements) was negatively associated with both blood and urinary Cd concentrations, even when dietary calcium and iron intakes were also considered [32]. Conversely, diets marginal in iron, calcium, and zinc were related to higher intestinal absorption (40% vs. 20% in controls) and tissue retention of Cd in experimental animals [33], although it is unclear whether this was due to the low nutrient content of the diet or to nutritional deficiencies that result from long-term consumption of nutrient-poor diets.

A number of studies have estimated the potential exposure to Cd from the total diet and specific foods; however, there is little understanding of how the typical diet is related to Cd concentrations in blood or urine. Few studies have investigated the extent to which dietary intakes predict body Cd concentrations among women of reproductive age, and pregnant women in particular. The aim of this study was to examine diet (food groups and dietary patterns) as a predictor of B-Cd in pregnant women participating in the UK Avon Longitudinal Study of Parents and Children (ALSPAC).

## 2. Materials and Methods

### 2.1. The ALSPAC Study

The sample was derived from ALSPAC, a population-based study investigating environmental and genetic influences on the health, behaviour, and development of children. This database includes a large number of participants and a wide range of social and demographic information to enable the most appropriate selection of covariates. All pregnant women in the former Avon Health Authority with an expected delivery date between 1 April 1991 and 31 December 1992 were eligible for the study. In total, 14,541 pregnant women were enrolled. The social and demographic characteristics of this cohort were similar to those found in UK national census surveys [34,35]. Further details of ALSPAC are found at [36]. The study website contains details of all the data that are available through a fully searchable data dictionary and variable search tool [37]. Ethics approval for the study was obtained from the ALSPAC Ethics and Law Committee and the Local Research Ethics Committees. Informed consent for the use of data collected via questionnaires and clinics was obtained from participants following the recommendations of the ALSPAC Ethics and Law Committee at the time.

### 2.2. Exposures

#### 2.2.1. Food Frequency Questionnaires

The dietary intakes were collected from a food frequency questionnaire (FFQ) sent to the ALSPAC mothers at 32 weeks’ gestation. The FFQ asked about the current frequency of consumption of 43 different foods and drinks. Possible answers were: (i) never or rarely; (ii) once in 2 weeks; (iii) one to three times per week; (iv) four to seven times per week; (v) more than once per day. This FFQ has been shown to produce mean nutrient intakes [38] similar to those obtained for women in the UK National Diet and Nutritional Survey for adults [39]. The specific question on the frequency of oily fish consumption has also been validated by comparison with the erythrocyte fatty acid composition of pregnancy blood samples: the erythrocyte docosahexaenoic acid (DHA) content increased significantly with an increasing frequency of consumption of oily fish (*p* < 0.001) [40].

#### 2.2.2. Food Groups

We combined individual foods of interest into 11 food groups: (1) meats; (2) fish; (3) pulses; (4) nuts; (5) soya bean products; (6) root vegetables; (7) leafy greens and green vegetables; (8) breads and cereals; (9) cakes and biscuits; (10) pastas and rice; (11) pies/pastries. The combinations of foods are described in Table S1. According to the originally reported frequency of consumption per type of food (categories i–v, above), mothers were then allocated to frequency categories for the new combined food groups, using the original categories provided as part of the FFQ. When consumption varied by food type, mothers were allocated to the subcategory representing the most frequent consumption. For example, within “meats” where a mother reported the consumption of poultry to be “one to three times per week” and red meat to be “never or rarely”, she would be assigned to “one to three times per week” for the combined “meats” group.

#### 2.2.3. Dietary Patterns

Principal components analysis (PCA) was used to derive underlying dietary patterns in this population. This has been described in detail elsewhere [41]. Briefly, the number of components best representing the data was chosen on the basis of a scree plot and the interpretability of the patterns. A component score was created for each woman for each of the components identified, calculated by multiplying the factor loadings by the corresponding standardised value for each food and summing across the food items (see Northstone, Emmett, and Rogers [41] for factor loadings and variance explained). Each score has a mean of 0 and a higher score indicates closer adherence to that dietary pattern. Five components were obtained: ‘health conscious’ (high factor loadings for salad, fruit, rice, pasta, oat and bran-based breakfast cereals, fish, pulses, fruit juices, and non-white bread); ‘traditional’ (high consumption of all types of vegetables and red meat and poultry); ‘processed’ (high intakes of high-fat processed foods, such as meat pies, sausages and burgers, fried foods, pizza, chips, and baked beans); ‘confectionery’ (high intakes of foods with high sugar content, such as chocolate, sweets, biscuits, cakes, and other puddings), and ‘vegetarian’ (high loadings for meat substitutes, pulses, nuts, and herbal teas, and high negative loadings for red meat and poultry).

### 2.3. Outcomes

#### 2.3.1. Collection, Storage, and Analysis of Blood Samples

Whole blood samples were collected in acid-washed vacutainers (Becton and Dickinson, Oxford, UK) by midwives as early as possible in pregnancy (median of 11 weeks’ gestation (IQR 9–13 weeks) with a mode of 10 weeks). Whole blood samples were stored in the original tube at 4 °C at the collection site before being transferred to the central Bristol laboratory within 1–4 days. Samples were at ambient temperature during transfer (up to 3 h). They were then stored at 4 °C until analysis. Details of the analysis have been reported previously [42]. In brief, inductively coupled plasma mass spectrometry in standard mode (R. Jones, Centers for Disease Control and Prevention (CDC), Bethesda, MD, USA; CDC Method 3009.1) was used to measure B-Cd with appropriate quality controls. The analyses were completed for 4286 women. In total, 1119 samples had a Cd concentration below the limit of detection (LOD) (0.20 µg/L): these were assigned a value of 0.14 µg/L (LOD/√2) to reflect the log-normal distribution [43,44]. In total, 4211 women with B-Cd data also had data on gestational age at the time of sampling.

#### 2.3.2. Potential Confounders

Potential confounding factors were defined a priori from the literature and included measures of socio-economic positioning (SEP), body mass index (BMI), estimated energy intake, lifestyle indicators (alcohol consumption and smoking status before and during pregnancy), and haemoglobin concentrations. Indicators of SEP were: age at pregnancy (categorised as ≤19, 20–24, 25–29, 30–34, ≥35 years), highest level of educational attainment (none/Certificate of School Education, vocational/Ordinary level, Advanced level and above), and Townsend score, a measure of material deprivation based on geographical area, incorporating census-based data on unemployment, non–car ownership, non-home ownership, and household overcrowding (quartiles from least deprived to most deprived) [45]. Data on BMI (based on height and pre-pregnancy weight), smoking status (yes/no), and alcohol consumption (yes/no) during the first trimester and smoking status pre-pregnancy (yes/no) were obtained from questionnaires completed during pregnancy. Energy intake was estimated from the FFQ at 32 weeks’ gestation [46]. Haemoglobin concentrations were extracted from obstetric clinic records (the first recorded concentration was used to correspond with the gestational time of the blood sample for Cd analysis).

### 2.4. Statistical Analysis

Statistical analysis was conducted using STATA version 14.2 (StataCorp, College Station, TX, USA).

We initially examined potential response bias in the sample by comparing mothers who completed the FFQ and had available data on B-Cd with those who did not. The associations between B-Cd and background characteristics were examined.

Continuous B-Cd results were transformed into a binary outcome variable by splitting the sample at the statistical median of 0.26 µg/L to address the skewed nature of the data. Univariable and multivariable logistic regression modelling were used to examine the likelihood of having B-Cd ≥ median of 0.26 µg/L in cases with complete exposure, outcome, and confounder data only. Two sets of models were produced, whereby one set included the dietary pattern scores as separate predictors, and a second set, the individual food groups. Dietary pattern scores and food groups were not mutually adjusted for. A number of potential confounders were considered in the adjusted models: indicators of SEP, BMI, energy intake, smoking status in the first trimester and alcohol consumption, and haemoglobin concentrations. There was a decline in the prevalence of smoking of 7.6 percentage points from pre-pregnancy to the first trimester (prevalence of smoking in the first trimester was 22.2%). B-Cd largely reflects recent exposure (previous 2–3 months) although it may also include a contribution from longer term exposure [47]: as the blood samples were obtained at a median of 11 weeks, only smoking in the first trimester (which broadly represents the previous 2–3 months) was used as a confounder in the main adjusted analyses. Results are reported as unadjusted and adjusted odds ratios (OR) with 95% confidence intervals (CI).

### 2.5. Sensitivity Analyses

We also conducted three sensitivity analyses by repeating all descriptive statistics as well as logistic regression modelling on subsets of the complete case sample: (1) only mothers who did not smoke in the first trimester; (2) only mothers who did not smoke in the first trimester and did not smoke immediately before becoming pregnancy; and (3) only mothers with B-Cd ≥ LOD.

## 3. Results

### 3.1. Diet and Blood Cd Concentrations in Pregnant Women in the Main Analysis (Overall Sample)

**Figure 1:**
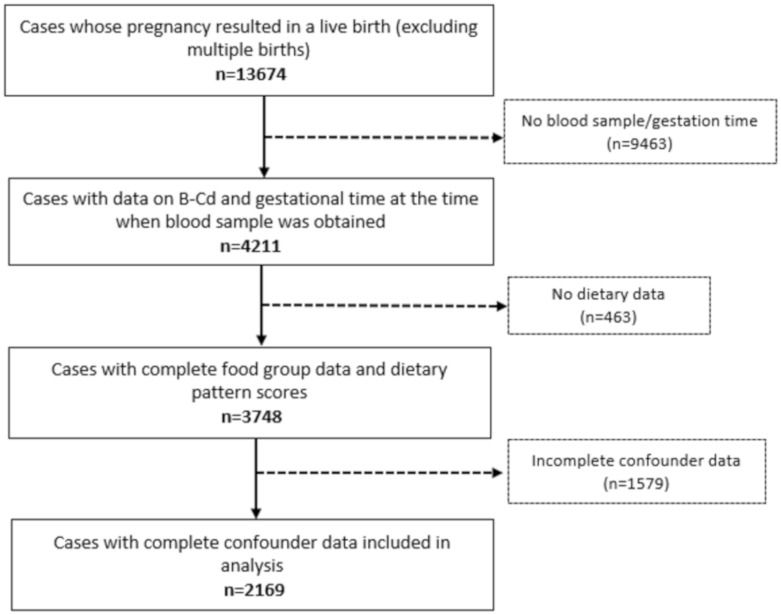
Flow chart for selection of participants from the UK ALSPAC cohort.

The derivation of the sample included in the analysis of this study is summarised in the flow chart (Figure 1). Table 1 summarises the included and excluded sample in terms of sociodemographic and lifestyle characteristics. Compared with excluded participants, those included in the analysis were more likely to be older, have higher educational attainment, live in less deprived geographical areas as indicated by the Townsend score, and were less likely to be smokers in the first trimester.

The mean B-Cd concentration was 0.50 (SD 0.58), range 0.14–6.30, with a median value of 0.26 (IQR 0.14–0.54) µg/L, but 664 (31%) women had values below the LOD. The mean value for ‘never vegetarian’ was 0.50 (SD 0.58), ‘vegetarian in the past’ 0.57 (SD 0.66), and ‘presently vegetarian’ 0.42 (SD 0.41) µg/L (*p* = 0.384 for ‘never vegetarian’ vs. ‘presently vegetarian’).

Participant characteristics according to the B-Cd category (< median or ≥ median) are shown in Table 2. Compared with participants with B-Cd < median, those with B-Cd ≥ median were more likely to be younger, have lower educational attainment, reside in more deprived geographical areas (Townsend score), and be smokers.

Only one of the five dietary patterns showed evidence of an association with B-Cd (Table 3). Closer adherence to the *health conscious* pattern indicated a reduced likelihood of having B-Cd ≥ median in the unadjusted model (model 1: OR 0.27 (95% CI 0.21–0.35) for quartile 4 vs. quartile 1) as well as the adjusted model (model 2: 0.56 (0.39–0.81)). Women in the highest quartile of the *processed* pattern had a higher likelihood of having B-Cd ≥ median (model 1: 1.99 (1.55–2.54)), but this association was attenuated in the adjusted model (model 2: 1.19 (0.84–1.68)). Similarly, having a *vegetarian* pattern was associated with a greater likelihood of B-Cd ≥ median (model 1: 1.51 (1.19–1.92) that was attenuated after adjustment (1.25 (0.93–1.68)). No other dietary patterns were associated with B-Cd in the unadjusted or adjusted models.

In the analysis of the food groups (Table 4), two categories were negatively associated with B-Cd in the unadjusted and adjusted models: (1) compared with consumption ≤1 to 3 times per week, eating *all leafy greens and green vegetables* ≥4 times per week was negatively associated with B-Cd in the unadjusted (model 1: 0.62 (0.51–0.76)) and adjusted (model 2: 0.72 (0.56–0.92)) analyses; (2) compared with *all meats* < once in 2 weeks, consumption ≥4 times per week was negatively associated with B-Cd in the unadjusted (model 1: 0.62 (0.47–0.83)) and adjusted (model 2: 0.66 (0.46–0.95)) model. The was a trend for a negative association of increasing all fish consumption with B-Cd. There was evidence of a negative association between the consumption of *root vegetables* and B-Cd in the unadjusted analysis comparing never/rarely vs. ≥ 4 times per week (model 1: 0.42 (0.27–0.65)). However, it was attenuated on adjustment (model 2: 0.77 (0.44–1.34)). Similarly, we observed negative associations between B-Cd and *bread and cereal*, *cakes and biscuits*, *pasta and rice*, *all pulses*, and *all nuts* in the unadjusted but not in the adjusted models.

### 3.2. Sensitivity Analysis 1: Diet and Blood Cd Concentrations in Pregnant Women with Exclusion of Those Who Smoked in the First Trimester

The demographic characteristics of participants who did not smoke in the first trimester by B-Cd were similar to those in the main analysis: compared with participants with B-Cd < median, those with B-Cd ≥ median were more likely to be younger, have lower educational attainment, and reside in more deprived geographical areas (Townsend score) (Table S2). When participants who smoked in the first trimester were excluded, the results were broadly similar to those of the whole group. The *health conscious* dietary pattern again predicted a likelihood of having B-Cd < median in the and adjusted models, but the *processed* pattern was not associated even in the unadjusted model (Table S3). *All meats*, *all fish*, and *all leafy green and green vegetables* were again associated with a lower likelihood of having B-Cd ≥ median in both the unadjusted and adjusted models. *Breads and cereals*, *cakes and biscuits*, and *pasta and rice* consumption were also negatively associated with the likelihood of having higher B-Cd in the unadjusted models but not in the adjusted models (Table S4).

### 3.3. Sensitivity Analysis 2: Diet and Blood Cd Concentrations in Pregnant Women with Exclusion of Those Who Smoked in the First Trimester and Pre-Pregnancy

With the additional exclusion of those who smoked pre-pregnancy, the demographic characteristics of participants by B-Cd showed similar but weaker associations compared with only the exclusion of participants who smoked in the first trimester: Compared with participants with B-Cd < median, those with B-Cd ≥ median were more likely to be younger and reside in more deprived geographical areas (Townsend score). However, there was no association with lower educational attainment (Table S5). The results of the analyses of the association with dietary patterns and foods/food groups were broadly similar to those of the whole group and the exclusion only of those who smoked in the first trimester: The *health conscious* dietary pattern again predicted a likelihood of having B-Cd < median in the unadjusted and adjusted models, but there were no other associations (Table S6). Negative associations for *all fish* were again evident but not for *all meat* or *all leafy green and green vegetables* (Table S7).

### 3.4. Sensitivity Analysis 3: Diet and Blood Cd Concentrations Among Pregnant Women with Detectable B-Cd

The demographic characteristics of participants with detectable B-Cd were similar to the those in the complete case analysis and to non-smokers: those with B-Cd ≥ median were more likely to be younger, have lower educational attainment, and reside in more deprived geographical areas (Townsend score) (Table S8). Associations with dietary patterns and food/food groups were generally consistent with the main findings. The *health conscious* pattern was associated with a lower likelihood of B-Cd ≥ median and the *processed* pattern with a higher likelihood, but these associations were attenuated on adjustment. The *confectionery* pattern was associated with a lower likelihood of B-Cd ≥ median, whereas the *vegetarian* pattern was associated with a greater likelihood in both the unadjusted and adjusted models (Table S9). This was not explained by vegetarians being more likely to have B-Cd > LOD (Table S10). *All cakes and biscuits* and *all pies and pastries* were associated with a lower likelihood of having B-Cd ≥ median in both the unadjusted and adjusted models (Table S11).

## 4. Discussion

Diet is an important route of exposure to Cd in pregnancy for non-smokers, but there has been little work on the dietary predictors of B-Cd in pregnant women. We found that a *health conscious* dietary pattern was associated with a lower likelihood of B-Cd ≥ median value in a group of pregnant women in the UK. There was a similarly reduced likelihood of B-Cd ≥ median value for the food group *all leafy green and green vegetables*, which appears consistent with the *health conscious* pattern, and with the food group *all meats*.

Cd is a persistent environmental pollutant that is readily incorporated into plant tissues during cultivation [30], and hence into meat and meat products and into dairy foods by bioaccumulation. Foods and food products can also be subject to external contamination (from dust, for example) during growth and processing [1,2]. Following exposure through food consumption and from other sources, Cd accumulates in all tissues, with the greatest concentrations in the liver and kidney [30]. The overall half-life is >26 years, with excretion through faeces and urine [5]. In pregnancy, the placenta acts as a partial barrier to Cd [10,48].

The most frequently used biomarkers for Cd exposure are whole blood or urine concentrations. B-Cd primarily indicates short-term exposure over 2–3 months at low to moderate exposures; urine concentration reflects longer-term Cd storage, particularly in the kidney [49]. Exposure to Cd was measured as B-Cd in this study: the concentration in this population (for complete cases: median 0.26 (IQR 0.14–0.54), mean 0.50 (SD 0.58), and geometric mean 0.33 µg/L) was similar to recent concentrations from pregnant women in similar developed countries [4] (for example, USA: geometric mean 0.18 µg/L; Sweden: mean 0.30 µg/L) [50,51]. Cigarette smoking is the main non-dietary predictor of B-Cd in populations not exposed through occupation [52]. Disparities in blood concentrations between cohorts may therefore indicate differences in smoking prevalence as well as differences in dietary intakes. Cohorts in which the prevalence of smoking is high tend to have high B-Cd. For example, in a French cohort in which 28% of women smoked during pregnancy and 30% experienced passive smoking, the median B-Cd was 0.8 (IQR 0.1–4.6) µg/L [53]. There are no national or international guidelines for maximum B-Cd specifically for pregnancy to our knowledge. The German Human Biomonitoring Commission for all non-smoking adults indicates a population reference value of 1 µg/L [54]. In total, 98% of our non-smoking complete case sample were below this concentration. In our sample, the relative contribution of foods and food groups to B-Cd was similar in smokers and non-smokers: upon excluding smokers from our cohort (22% of sample with complete data), we found broadly similar results for the associations of dietary patterns, foods, and food groups with B-Cd to those in the whole sample.

The mean dietary intake of Cd in the UK has been estimated at 1.54 (95% CI 1.49–1.54) µg/per kg body weight per week, with 17.1% (95% CI 15.8–18.5) of individuals estimated to exceed the European Food Standards Agency’s current tolerable weekly intake of 2.5 µg/kg body weight per week [55]. Rice, bread and cereals, leafy vegetables, and some roots and tubers (carrots and potatoes) are relatively high in Cd on a weight for weight basis in the UK [56], and in the USA, cereals/bread, leafy vegetables and potatoes were the top three foods/food groups contributing to dietary Cd intake [57]. Potatoes were identified as having a high median Cd concentration in northern Italy (10.5 µg/kg) [58], and in Bangladesh, leafy green vegetables have been reported as having even higher Cd concentrations than root vegetables [20]. In contrast, we found that greater *all leafy green and green vegetables* consumption was associated with lower B-Cd, as was following a *health conscious* dietary pattern rich in plant-based foods, and there was no association with *root vegetable* intake. It is possible that the high levels of dietary fibre provided by the *health conscious* dietary pattern could reduce the relative absorption of Cd though a faster gut transit time, or possibly by changes in the gut microbiome. However, in assessing the contribution to overall dietary exposure to Cd, it is important to consider not only absolute concentrations but also the frequency of consumption and portion sizes. For example, for potatoes containing 10.5 µg Cd/kg, an average portion size of 175 g twice per week would contain 3.7 µg Cd, equivalent to only 2% of the UK’s tolerable weekly intake, assuming a woman weighs 60 kg. Given the relative absorption of Cd of ≤11% [5], the contribution of any particular food item to Cd exposure is likely to be relatively low even if its absolute Cd content is high.

Similar to *all leafy green and green vegetables*, greater consumption of the food group *all meats* was associated with a reduced likelihood of B-Cd ≥ median value. Some studies have found lower Cd concentrations in meats than in vegetables or cereals [20,58], but offal meats, such as kidney and liver, were high in Cd in China [59]. This latter finding was confirmed in an exposure assessment study based on food items from 14 European countries in which offal from farmed animals contained a mean of 316 µg Cd/kg [56], although offal would be a rarely consumed food item in most European-style diets. As noted earlier, for some individuals and population groups, meat consumption contributes little to daily Cd exposure because of the consumption of relatively low amounts of meat. Among adults from Bangladesh, for example, the estimated daily intake of Cd from the diet was 35 µg/day, of which only 2% came from meats/fish, with much greater contributions from green vegetables (35%) and from steamed rice (54%) [20]. Similarly, among Mexican women, the contribution of meat and poultry to daily Cd consumption was low compared with the contribution of vegetables, grains, corn, and potatoes [23]. Among adults from northern Italy, whose average diet is about 8% meats, 10% vegetables, and 12% grains by weight, meat also contributed only a small amount (IQR 0.05–0.12 µg/day) to the estimated daily Cd exposure [58]. Similarly, meat was not amongst the main contributors to dietary Cd intake in the USA [57].

It might be expected that in non-vegetarians, the high iron content of meats would result in better iron status and thus lower competition for absorptive binding sites (DMT1), resulting in lower relative Cd absorption, and hence lower B-Cd concentrations compared with vegetarians. In support of this, concentrations of Cd were significantly higher in pregnant women with low plasma ferritin at 18 weeks’ gestation in a Norwegian study that included smokers and non-smokers [60]. Although we did not find any difference in B-Cd among women who reported consuming vegetarian diets compared with those who did not consume vegetarian diets, only 6% of women reported that they were vegetarian at present. In the main regression analysis, there was a suggestion of an association between the *vegetarian* pattern and B-Cd in the unadjusted analysis, although this was attenuated on adjustment. However, in the sensitivity analysis when values < LOD were excluded, there was a strong association with the *vegetarian* pattern. Non-vegetarians from the Slovak Republic had B-Cd of about 25% that of vegetarians [61], although this was related to higher consumption of wholegrain products rather than vegetables. It is possible that meat consumption could be proxy for another protective factor. However, both our study and that of Krajcovicova–Kudladkova included a relatively small number of women identifying themselves as vegetarian (*n* = 131 and *n* = 80, respectively), which may reduce the reliability of the results in vegetarians in both studies.

Calcium, of which milk and other dairy products are a major dietary source, is a low affinity inhibitor of DMT1 activity [62] and so a diet that is high in calcium might be expected to lower B-Cd. We found no association of B-Cd with the frequency of drinking milk or with calcium intake, although a previous study of East and South Asian women of reproductive age who had recently immigrated to Canada (≤5 years) found that higher dairy product consumption was associated with a 14% lower risk of elevated B-Cd [63]. In addition, increasing dietary calcium and vitamin D in pregnant women also in Canada was associated with lower B-Cd [64]. The disparity between our study and these two studies could be accounted for by differences in the mean frequency of milk/dairy consumption, but comparison is difficult because of differences in the way in which variables are expressed [63] included data on the consumption of dairy items (52 ± 34 times/month); the present UK study included data specifically for milk (44% never or rarely consumed milk, 48% had 7–14 glasses per week, and 9% had ≥21 glasses per week). The disparity in the results could also indicate differences in the frequency of consumption of other foods and food groups, which could have a secondary effect on both dietary intake and absorption of Cd.

This study has several strengths. First, there are many advantages of using dietary patterns and food groups: (1) they take into account the effects of combinations of nutrients and foods; (2) they can provide information that is more meaningful in translation to public health messages; and (3) identification of dietary patterns by PCA is less sensitive to inaccuracy and bias in the dietary data collection than is the assessment of single nutrients [65]. Second, the study provides a valuable addition to the body of literature on Cd and dietary patterns by including a relatively large population of pregnant women, a vulnerable group for whom it is important to reduce Cd exposure to a minimum. To our knowledge, these are the most recent data on Cd exposures in pregnant women in the UK. It is likely that the prevalence of smoking will have declined, thus reducing exposures for some women. Third, in addition to dietary patterns, we were able to look further at specific foods and food groups that might have contributed to increased B-Cd (either because of being sources of cadmium, and/or because they provided nutrients that interfered with cadmium absorption or metabolism). Fourth, we accounted for smoking status, which is a major source of Cd exposure. Smoking prevalence was 29.8% prior to pregnancy and 22.2% in the first trimester. In regression models conducted on the full study sample, we added first trimester smoking status as a covariate. We also conducted sensitivity analyses by excluding first trimester smokers and then first trimester smokers plus those who smoked pre-pregnancy. Both approaches yielded similar findings.

There are also some limitations. First, the study was based in a largely urban population in the UK and may have limited generalisability to other populations of pregnant women both in the UK and in other countries. Second, Cd exposure was relatively low compared with the German recommended population upper limit [54] and this may also limit generalisability to countries with higher levels. Third, there were a large number of values for blood Cd concentrations below the limit of detection, making the use of the measurement as a continuous variable in linear models less robust. However, we used a well-documented means of correcting values below the limit of detection. We also modelled B-Cd as a categorical variable to minimise the influence of values < LOD on the results. Fourth, blood was sampled for Cd analysis in the first trimester, whereas the dietary data were collected in the third trimester. The difference in timing for these assessments could make it difficult to infer causation. However, it is important to point out that B-Cd reflects Cd exposure over the previous 2–3 months, which could reduce the effect of the time lag between the blood and dietary data collections. In addition, both B-Cd and urine Cd are stable during pregnancy [66]. Dietary patterns in pregnancy have also been shown to be stable [67], as have intakes of energy and macronutrients [68,69,70]. Dietary patterns specifically in pregnancy in ALSPAC are similar to those at 4 years postpartum [71]. Thus, it is likely that the data in the present study are representative of diet and biomarker concentrations throughout pregnancy, strengthening the plausibility of inference of causation. It is also possible that the consumption of vitamin and mineral supplements, which could affect Cd absorption, were imperfectly captured by the food frequency questionnaire. Fifth, misreporting (both under- and over-reporting) are inevitable in dietary data collection and this can undermine the validity of associations. Although there are established methods to identify misreporting of energy, those for foods and food groups are much less well understood. Sixth, the sub-sample of women included in this study were not completely identical in sociodemographic characteristics to those that were excluded. It is possible that they had higher B-Cd than those who were excluded, and this would drive the results toward the null (no associations). Finally, in those who recently stopped smoking (either before pregnancy or very early in pregnancy) and were classified as not smoking in the first trimester, it is possible that B-Cd was elevated by the effect of previous exposure through smoking. This could have the effect of causing misclassification bias. While it is known that current smokers have less healthy dietary patterns than non-smokers [72], supporting the use of current smoking as a confounder in the present analyses, little is known about the effect of smoking cessation on dietary patterns so it was not possible to take this into account in the analyses.

## 5. Conclusions

In a group of UK pregnant women, a *health conscious* dietary pattern (characterised by high factor loadings for salad, fruit, rice, pasta, oat and bran-based breakfast cereals, fish, pulses, fruit juices, and non-white bread) was associated with a lower likelihood of B-Cd being ≥ median value. There were no associations for any food or food group with B-Cd in the adjusted models, with the exception of a negative association for *all leafy green and green vegetables*, which appears consistent with the *health conscious* pattern, and with *all meats*. This study provides evidence to support a healthy and varied diet in pregnancy to minimise B-Cd, incorporating foods from all food groups in accordance with national recommendations.

## Figures and Tables

**Table 1:** Included vs. excluded sample comparison: pregnant women enrolled in ALSPAC with complete exposure, outcome, and confounder data (n = 2169) by indicators of socioeconomic positioning and other background characteristics compared with the rest of the ALSPAC cohort vs. excluded sample.

Participant Characteristic	n (%)		Odds Ratio (95% CI)	*p* Value
	Excluded	Included		
	115054 (84.2)	2169 (15.9)		
Maternal age at pregnancy (years)				
≤19	600 (5.2)	53 (2.4)	1.00	
20 to <25	2341 (20.4)	313 (14.4)	1.51 (1.11, 2.05)	
25 to <30	4437 (38.6)	850 (39.2)	2.17 (1.62, 2.90)	
30 to <35	3014 (26.2)	709 (32.8)	2.67 (1.80, 3.58)	
≥35	1113 (9.7)	242 (11.2)	2.46 (1.80, 3.37)	<0.001
Maternal education				
None/CSE	3104 (31.1)	541 (24.9)	1.00	
Vocational/O-level	5672 (56.9)	1262 (58.2)	1.27 (1.14, 1.42)	
A-level and above	1191 (12.0)	366 (16.9)	1.76 (1.52, 2.05)	<0.001
Maternal social class				
I/II	2936 (36.9)	734 (39.2)	1.00	
III non-manual/III manual	4041 (50.8)	936 (50.0)	0.93 (0.83, 1.03)	
IV/V	982 (12.3)	203 (10.8)	0.83 (0.70, 0.98)	0.074
Paternal social class				
Total n				
I/II	3850 (44.3)	964 (48.3)	1.00	
III non-manual/III manual	3733 (42.9)	795 (39.8)	0.85 (0.77, 0.94)	
IV/V	1115 (12.8)	239 (12.0)	0.86 (0.73, 1.00)	0.055
Townsend score				
1	2347 (32.5)	658 (30.3)	1.00	
2	1398 (19.4)	341 (15.7)	0.87 (0.75, 1.01)	
3	1907 (26.44)	578 (26.7)	1.08 (0.95, 1.23)	
4	1560 (21.63)	592 (27.3)	1.35 (1.19, 1.54)	<0.001
Smoking status in 1st trimester				
No	7973 (74.4)	1687 (77.8)	1.00	
Yes	2737 (25.6)	482 (22.2)	0.83 (0.75, 0.93)	0.001
Maternal dietary scores				
Health conscious	−0.022 ± 1.004	0.100 ± 0.975		<0.001
Traditional	−0.002 ± 1.006	0.009 ± 0.217		0.531
Processed	0.013 ± 1.012	−0.056 ± 0.942		0.006
Confectionery	0.006 ± 1.009	−0.022 ± 0.960		0.200
Vegetarian	−0.003 ± 0.994	0.010 ± 1.028		0.643

Educational attainment: none/Certificate of School Education, vocational/Ordinary level, Advanced level and above. Townsend score is a measure of deprivation: 1 is the least deprived, score 4 the most deprived [45]. Social class: I, higher managerial, administrative or professional; II, intermediate managerial, administrative or professional; III non-manual, supervisory or clerical and junior management, administrative or professional; III manual, skilled manual workers; IV, semi-skilled and unskilled manual workers; V, casual or lowest grade workers

**Table 2:** Blood cadmium concentrations in pregnant women enrolled in ALSPAC by indicators of socio-economic positioning and lifestyle (complete case analysis including smokers, *n* = 2169).

Variable	Included Sample Observations *n* (%)	*n* (%)		Odds Ratio (95% CI)	*p* Value
		B-Cd < Median	B-Cd ≥ Median		
Maternal age (years)					
≤19	53 (2.4)	15 (1.2)	38 (3.6)	1.00	
20 to <25	313 (14.4)	126 (10.4)	187 (19.5)	0.59 (0.3, 1.1)	
25 to <30	850 (39.2)	480 (39.7)	370 (38.5)	0.30 (0.2, 0.6)	
30 to <35	711 (32.8)	444 (36.7)	267 (27.8)	0.24 (0.1, 0.4)	
≥35	242 (11.7)	144 (11.9)	98 (10.2)	0.27 (0.1, 0.5)	<0.001
**Maternal education**					
None/CSE	541 (24.9)	231 (19.1)	310 (32.3)	1.00	
Vocational/O-level	1262 (58.2)	714 (59.1)	548 (57.1)	0.57 (0.5, 0.7)	
A-level and above	366 (16.9)	264 (21.8)	102 (10.6)	0.29 (0.2, 0.4)	<0.001
**Townsend score**					
1	658 (30.3)	420 (34.7)	238 (24.8)	1.00	
2	341 (15.7)	208 (17.2)	133 (13.9)	1.13 (0.9, 1.5)	
3	578 (26.7)	315 (26.1)	263 (27.4)	1.47 (1.2, 1.9)	
4	592 (27.3)	266 (22.0)	326 (34.0)	2.16 (1.7, 2.7)	<0.001
**Smoking status in 1st trimester**					
No	1687 (77.8)	1188 (98.3)	499 (52.0)	1.00	
Yes	482 (22.2)	21 (1.74)	461 (48.0)	52.3 (33.3, 82.0)	<0.001
**Smoked regularly pre-pregnancy**					
No	1522 (70.2)	1051 (96.5)	471 (43.6)	1.00	<0.001
Yes	647 (29.8)	38 (0.3)	609 (56.4)	28.6 (20.7, 39.4)	
**Alcohol consumption**					
No	959 (44.2)	548 (45.3)	411 (42.8)	1.00	
Yes	1210 (55.8)	661 (54.7)	549 (57.2)	1.11 (0.9, 1.3)	0.241
**BMI**					
Normal/underweight	1711 (78.9)	967 (80.0)	244 (77.5)	1.00	
Overweight	343 (15.8)	185 (15.3)	158 (16.5)	1.11 (0.9, 1.4)	
Obese	115 (5.3)	57 (4.7)	58 (6.04)	1.32 (0.9, 1.9)	0.268
**Vegetarian diet**					
Never	1812 (85.3)	1024 (86.6)	788 (83.7)	1.00	
In the past	180 (8.5)	87 (7.4)	93 (9.9)	1.39 (1.0, 1.9)	
Present	131 (6.2)	71 (6.0)	60 (6.4)	1.10 (0.8, 1.6)	0.104

Educational attainment: none/Certificate of School Education, vocational/Ordinary level, Advanced level and above. Townsend score is a measure of deprivation: 1 is the least deprived, score 4 the most deprived [45]. Social class: I, higher managerial, administrative or professional; II, intermediate managerial, administrative or professional; III non-manual, supervisory or clerical and junior management, administrative or professional; III manual, skilled manual workers; IV, semi-skilled and unskilled manual workers; V, casual or lowest grade workers. Median B-Cd 0.26 µg/L. BMI (body mass index): normal/underweight <24.9; overweight 25.0–29.9; obese ≥30.0 kg/m^2^.

**Table 3:** Associations of dietary patterns with blood cadmium concentrations in pregnant women enrolled in ALSPAC (complete case analysis including smokers, *n* = 2169).

Pattern	Quartile	Median B-Cd (µg/L)	*n* (%)		Unadjusted Model 1	Adjusted Model 2
			B-Cd < Median	B-Cd ≥ Median		
		Group median: 0.26				
Health conscious	1	0.40	173 (14.3)	285 (29.7)	1.00	1.00
	2	0.29	262 (21.7)	260 (27.1)	0.60 (0.47–0.78)	0.86 (0.62–1.18)
	3	0.25	362 (29.9)	232 (24.2)	0.39 (0.30–0.50)	0.69 (0.50–0.96)
	4	0.22	412 (34.1)	183 (19.1)	0.27 (0.21–0.35)	0.56 (0.39–0.81)
					*p* trend <0.001	*p* trend = 0.001
Traditional	1	0.28	279 (23.1)	254 (26.5)	1.00	1.00
	2	0.25	302 (24.9)	209 (21.8)	0.76 (0.600.97)	0.81 (0.60–1.09)
	3	0.27	314 (26.0)	252 (26.3)	0.88 (0.70–1.12)	0.88 (0.66–1.18)
	4	0.26	314 (26.0)	245 (25.5)	0.86 (0.68–1.09)	0.94 (0.70–1.27)
					*p* trend = 0.408	*p* trend = 0.903
Processed	1	0.23	362 (29.9)	205 (21.4)	1.00	1.00
	2	0.25	338 (28.0)	231 (24.1)	1.21 (0.95–1.53)	1.07 (0.81–1.43)
	3	0.29	266 (22.0)	250 (26.0)	1.66 (1.30–2.12)	1.46 (1.08–1.97)
	4	0.31	243 (20.1)	274 (28.5)	1.99 (1.56–2.54)	1.19 (0.84–1.68)
					*p* trend <0.001	*p* trend = 0.078
Confectionery	1	0.28	289 (24.3)	122 (24.5)	1.00	1.00
	2	0.26	308 (25.9)	132 (26.5)	0.92 (0.731.16)	1.05 (0.79–1.41)
	3	0.25	313 (26.4)	130 (26.1)	0.81 (0.64–1.03)	1.07 (0.79–1.45)
	4	0.27	278 (23.4)	115 (23.1)	0.93 (0.73–1.19)	1.05 (0.74–1.48)
					*p* trend = 0.375	*p* trend = 0.844
Vegetarian	1	0.24	365 (30.2)	214 (22.3)	1.00	1.00
	2	0.27	303 (25.1)	240 (25.0)	1.35 (1.06–1.72)	1.13 (0.85–1.52)
	3	0.29	258 (21.3)	255 (26.6)	1.69 (1.32–2.15)	1.13 (0.83–1.53)
	4	0.28	283 (23.4)	251 (26.2)	1.51 (1.19–1.92)	1.25 (0.93–1.68)
					*p* trend <0.001	*p* trend = 0.132

Model 2 adjusted for maternal age, maternal education, Townsend score, BMI and energy intake, alcohol consumption and smoking status during first trimester, and haemoglobin concentrations.

**Table 4:** Associations of the frequency of intakes of foods and food groups with blood cadmium concentrations in pregnant women enrolled in ALSPAC (complete case analysis including smokers, *n* = 2169).

	*n* (%)		OR (95% CI)	
	B-Cd < Median	B-Cd ≥ Median	Unadjusted Model 1	Adjusted Model 2
**Total *n***	1209	960		
**All meats combined**				
≤ Once in 2 weeks	148 (12.24)	151 (15.73)	1.00	1.00
≤3 times per week	752 (62.20)	613 (63.85)	0.80 (0.62–1.03)	0.77 (0.57–1.04)
≥4 times per week for at least one group	309 (25.56)	196 (20.42)	0.62 (0.47–0.83)	0.66 (0.46–0.95)
			*p* trend = 0.001	*p* trend = 0.021
**All fish**				
≤ Once in 2 weeks	545 (45.08)	537 (55.94)	1.00	1.00
≥1 to 3 times per week	614 (50.79)	391 (40.73)	0.65 (0.54–0.77)	0.76 (0.61–0.95)
≥4 to 7 times per week	50 (4.14)	32 (3.33)	0.65 (0.41–1.03)	0.82 (0.47–1.42)
			*p* trend <0.001	*p* trend = 0.026
**Milk (glasses per day) ^a^**				
None/rarely	513 (43.62)	406 (43.80)	1.00	1.00
1 to 2 glasses per day	473 (48.72)	433 (46.71)	0.95 (0.80–1.14)	0.83 (0.66–1.03)
≥3 glasses per day	90 (7.65)	88 (9.49)	1.24 (0.90–1.70)	0.86 (0.58–1.29)
			*p* trend = 0.548	*p* trend = 0.162
**All pulses combined**				
≤ Once in 2 weeks	1.37 (11.3)	135 (14.1)	1.00	1.00
≤3 times per week	960 (79.4)	750 (78.1)	0.79 (0.61–1.02)	0.74 (0.57–1.00)
≥4 times per week for at least one group	112 (9.3)	75 (7.8)	0.68 (0.47–0.99)	0.71 (0.45–1.12)
			*p* trend = 0.035	*p* trend = 0.105
**All nuts combined**				
Never/rarely	137 (11.3)	135 14.1	1.00	1.00
≤ Once in 2 weeks	960 (79.4)	750 78.1	0.79 (0.61–1.02)	0.74 (0.54–1.00)
≥1 to 3 times per week	112 (9.3)	75 7.8	0.68 (0.47–0.99)	0.71 (0.45–1.12)
			*p* trend = 0.009	*p* trend =0.875
**Soya bean products**				
Never or rarely	1096 (90.7)	870 90.6	1.00	1.00
≤ Once in 2 weeks	113 (9.4)	90 9.4	1.00 (0.75–1.34)	1.37 (0.97–1.92)
			*p* trend = 0.982	*p* trend = 0.051
**Root vegetables**				
Never or rarely	35 (2.9)	56 5.8	1.00	1.00
≤ One to 3 times per week per food	700 (57.9)	588 61.3	0.53 (0.34–0.81)	0.70 (0.41–1.22)
≥4 to 7 times per week	474 (39.2)	316 32.9	0.42 (0.27–0.65)	0.77 (0.44–1.34)
			*p* trend <0.001	*p* trend = 0.905
**All leafy green and green vegetables**				
≤1 to 3 times per week	236 (19.5)	269 28.0	1.00	1.00
≥4 times per week	973 (80.5)	691 72.0	0.62 (0.51–0.76)	0.72 (0.56–0.92)
			*p* trend <0.001	*p* trend = 0.005
**Combined breads and cereals**				
≤ Once a week	101 (8.35)	162 (16.88)	1.00	1.00
≤ One to 3 times per week per food	321 (26.55)	282 (29.38)	0.55 (0.41–0.74)	0.76 (0.53–1.10)
≥4 to 7 times per week	787 (65.10)	516 (53.75)	0.41 (0.31–0.54)	0.71 (0.50–1.01)
			*p* trend <0.001	*p* trend = 0.069
**All cakes and biscuits**				
≤ Once a week	183 (15.14)	205 (21.35)	1.00	1.00
≤ One to 3 times per week per food	600 (49.63)	471 (49.06)	0.70 (0.56–0.88)	0.79 (0.59–1.06)
≥4 to 7 times per week	426 (35.24)	284 (29.58)	0.59 (0.46–0.76)	0.79 (0.57–1.11)
			*p* trend <0.001	*p* trend = 0.204
**All pies and pastries**				
Never or rarely	245 (20.26)	196 (20.42)	1.00	1.00
≤ Once in 2 weeks	687 (56.82)	511 (53.23)	0.93 (0.75–1.16)	1.01 (0.77–1.32)
≥1 to 3 times per week	277 (22.91)	253 (26.35)	1.14 (0.89–1.47)	0.87 (0.62–1.21)
			*p* trend = 0.254	*p* trend = 0.343
**All pasta and rice**				
Never or rarely	89 (7.36)	125 (13.02)	1.00	1.00
≤ Once in 2 weeks	335 (27.71)	318 (33.13)	0.68 (0.49–0.92)	0.84 (0.56–1.24)
≥1 to 3 times per week	785 (64.93)	517 (53.85)	0.47 (0.35–0.63)	0.78 (0.53–1.15)
			*p* trend <0.001	*p* trend = 0.169

Model 2 adjusted for maternal age, maternal education, Townsend score + BMI and energy intake + alcohol consumption, and smoking status during first trimester + haemoglobin concentration. ^a^ A standard glass of milk is 200 mL. Calcium intake (quartiles): *p* for trend 0.690 in adjusted model (data not shown).

## Data Availability

Data are available to bona fide researchers on application to the ALSPAC Executive Committee.

## Supplementary Materials

The following are available online at https://www.mdpi.com/2072-6643/12/4/904/s1, Table S1: Food groups and individual foods combined for the analyses, Table S2: Sensitivity analysis 1: Blood cadmium concentrations in pregnant women enrolled in ALSPAC by indicators of socio-economic positioning and lifestyle (complete case analysis excluding smokers in the first trimester, *n* = 1687), Table S3: Sensitivity analysis 1: Associations of dietary patterns with blood cadmium concentrations in pregnant women enrolled in ALSPAC (complete case analysis excluding those who smoked in the first trimester, *n* = 1687), Table S4: Sensitivity analysis 1: Associations of frequency of intakes of foods and food group with blood cadmium concentrations in pregnant women enrolled in ALSPAC (complete case analysis excluding those who smoked in the first trimester, *n* = 1687); Table S5: Sensitivity analysis 2: Blood cadmium concentrations in pregnant women enrolled in ALSPAC by indicators of socio-economic positioning and lifestyle (complete case analysis excluding those who smoked in the first trimester and pre-pregnancy, *n* = 1518), Table S6: Sensitivity analysis 2: Associations of dietary patterns with blood cadmium concentrations in pregnant women enrolled in ALSPAC (complete case analysis excluding current smokers and pre-pregnancy smokers, *n* = 1518), Table S7: Sensitivity analysis 2: Associations of frequency of intakes of foods and food group with blood cadmium concentrations in pregnant women enrolled in ALSPAC (complete case analysis excluding those who smoked in the first trimester and pre-pregnancy, *n* = 1518), Table S8: Sensitivity analysis 3: Blood cadmium concentrations in pregnant women enrolled in ALSPAC by indicators of socio-economic positioning and lifestyle (complete case analysis excluding < LOD, *n* = 1505), Table S9: Sensitivity analysis 3: Associations of dietary patterns with blood cadmium concentrations in pregnant women enrolled in ALSPAC (complete case analysis excluding < LOD, *n* = 1505), Table S10: Blood Cd concentration in participants by vegetarian status in pregnant women enrolled in ALSPAC (in complete cases and in complete cases excluding those with values below the limit of detection), Table S11: Sensitivity analysis 3: Associations of frequency of intakes of foods and food group with blood cadmium concentrations in pregnant women enrolled in ALSPAC (complete case analysis excluding < LOD, *n* = 1505)

## Declarations

Ethics approval and consent to participate: Ethics approval for the study was obtained from the ALSPAC Ethics and Law Committee and the Local Research Ethics Committees. Informed consent for the use of data was collected via questionnaires and clinics was obtained from participants following the recommendations of the ALSPAC Ethics and Law Committee at the time.

## References

[1] Gao L., Chang J., Chen R., Li H., Lu H., Tao L., Xiong J. (2016). Comparison on cellular mechanisms of iron and cadmium accumulation in rice: Prospects in cultivating Fe-rich but Cd-free rice. Rice.

[2] McLaughlin M.J., Parker D.R., Clarke J.M. (1999). Metals and micronutrients—Food safety issues. Field Crops Res..

[3] Järup L., Åkesson A. (2009). Current status of cadmium as an environmental health problem. Toxicol. Appl. Pharmacol..

[4] Taylor C.M., Golding J., Emond A.M. (2014). Lead, cadmium and mercury levels in pregnancy: The need for international consensus on levels of concern. J. Dev. Orig. Health Dis..

[5] Faroon O., Ashizawa A., Wright S., Tucker P., Jenkins K., Ingerman L., Rudisill C., Inc S. (2012). Toxicologial Profile for Cadmium.

[6] Leazer T.M., Liu Y., Klaassen C.D. (2002). Cadmium absorption and its relationship to Divalent Metal Transporter-1 in the pregnant rat. Toxicol. Appl. Pharmacol..

[7] Kippler M., Goessler W., Nermell B., Ekström E.C., Lönnerdal B., El Arifeen S., Vahter M. (2009). Factors influencing intestinal cadmium uptake in pregnant Bangladeshi women—A prospective cohort study. Environ. Res..

[8] Sakamoto M., Chan H.M., Domingo J.L., Kubota M., Murata K. (2012). Changes in body burden of mercury, lead, arsenic, cadmium and selenium in infants during early lactation in comparison with placental transfer. Ecotoxicol. Environ. Saf..

[9] Gundacker C., Hengstschläger M. (2012). The role of the placenta in fetal exposure to heavy metals. Wien. Med. Wochenschr..

[10] Chen Z., Myers R., Wei T., Bind E., Kassim P., Wang G., Ji Y., Hong X., Caruso D., Bartell T. (2014). Placental transfer and concentrations of cadmium, mercury, lead, and selenium in mothers, newborns, and young children. J. Expo. Sci. Environ. Epidemiol..

[11] Luo Y., McCullough L.E., Tzeng J.Y., Darrah T., Vengosh A., Maguire R.L., Maity A., Samuel-Hodge C., Murphy S.K., Mendez M.A. (2017). Maternal blood cadmium, lead and arsenic evels, nutrient combinations, and offspring birthweight. BMC Public Health.

[12] Llanos M.N., Ronco A.M. (2009). Fetal growth restriction is related to placental levels of cadmium, lead and arsenic but not with antioxidant activities. Reprod. Toxicol..

[13] World Health Organization A Recipe for Safer Food. http://www.who.int/foodsafety/chem/TDS_recipe_2005_en.pdf.

[14] Callan A., Hinwood A., Devine A. (2014). Metals in commonly eaten groceries in Western Australia: A market basket survey and dietary assessment. Food Addit. Contam..

[15] Chen M.Y.Y., Chan B.T.P., Lam C.H., Chung S.W.C., Ho Y.Y., Xiao Y. (2014). Dietary exposures to eight metallic contaminants of the Hong Kong adult population from a total diet study. Food Addit. Contam..

[16] He P., Lu Y., Liang Y., Chen B., Wu M., Li S., He G., Jin T. (2013). Exposure assessment of dietary cadmium: Findings from Shanghainese over 40 years, China. BMC Public Health.

[17] Schwartz M.A., Lindtner O., Blume K., Heinemeyer G., Schneider K. (2014). Cadmium exposure from food: The German LExUKon project. Food Addit. Contam..

[18] Bannon D.I., Abounader R., Lees P.S., Bressler J.P. (2003). Effect of DMT1 knockdown on iron, cadmium, and lead uptake in Caco-2 cells. Am. J. Physiol. Cell Physiol..

[19] Spungen J.H. (2019). Children’s exposures to lead and cadmium: FDA Total Diet Study 2014–16. Food Addit. Contam. Part A Chem. Anal. Control Expo. Risk Assess..

[20] Al-Rmalli S.W., Jenkins R.O., Haris P.I. (2012). Dietary intake of cadmium from Bangladeshi foods. J. Food Sci..

[21] Amzal B., Julin B., Vahter M., Wolk A., Johanson G., Akesson A. (2009). Population toxicokinetic modeling of cadmium for health risk assessment. Environ. Health Perspect..

[22] Adams S.V., Quraishi S.M., Shafer M.M., Passarelli M.N., Freney E.P., Chlebowski R.T., Luo J., Meliker J.R., Mu L., Neuhouser M.L. (2014). Dietary cadmium exposure and risk of breast, endometrial, and ovarian cancer in the Women’s Health Initiative. Environ. Health Perspect..

[23] Moynihan M., Peterson K.E., Cantoral A., Song P.X.K., Jones A., Solano-Gonzalez M., Meeker J.D., Basu N., Tellez-Rojo M.M. (2017). Dietary predictors of urinary cadmium among pregnant women and children. Sci. Total Environ..

[24] Kim K., Nobles C., Purdue-Smithe A., Wactawski-Wende J., Pollack A., Freeman J., Alkhalaf Z., Andriessen V., Radoc J., Mumford S. (2019). Food intake and blood levels of mercury, lead, and cadmium among healthy reproductive aged women (P18-024-19). Curr. Dev. Nutr..

[25] Gunshin H., Mackenzie B., Berger U.V., Gunshin Y., Romero M.F., Boron W.F., Nussberger S., Gollan J.L., Hediger M.A. (1997). Cloning and characterization of a mammalian proton-coupled metal-ion transporter. Nature.

[26] Meltzer H.M., Branstæter A.L., Borch-Iohnsen B., Ellingsen D.G., Alexander J., Thomassen Y., Stigum H., Ydersbond T.A. (2010). Low iron stores are related to higher blood lead concentrations of manganese, cobalt and cadmium in non-smoking, Norwegian women in the HUNT2 study. Environ. Res..

[27] Kippler M., Ekström E.C., Lönnerdal B., Goessler W., Akesson A., El Arifeen S., Persson L.A., Vahter M. (2007). Influence of iron and zinc status on cadmium accumulation in Bangladeshi women. Toxicol. Appl. Pharmacol..

[28] Gallagher C.M., Chen J.J., Kovach J.S. (2011). The relationship between body iron stores and blood and urine cadmium concentrations in US never-smoking, non-pregnant women aged 20–49 years. Environ. Res..

[29] Meltzer H.M., Alexander J., Brantsæter A.L., Borch-Iohnsen B., Ellingsen D.G., Thomassen Y., Holmen J., Ydersbond T.A. (2016). The impact of iron status and smoking on blood divalent metal concentrations in Norwegian women in the HUNT2 Study. J. Trace Elem. Med. Biol..

[30] Satarug S. (2018). Dietary Cadmium intake and its effects on kidneys. Toxics.

[31] Vázquez M., Calatayud M., Jadán Piedra C., Chiocchietti G.M., Vélez D., Devesa V. (2015). Toxic trace elements at gastrointestinal concentration. Food Chem. Toxicol..

[32] Vance T.M., Chun O.K. (2015). Zinc intake is associated with lower cadmium burden in US adults. J. Nutr..

[33] Reeves P.G., Chaney R.L. (2008). Bioavailability as an issue in risk assessment and management of food cadmium: A review. Sci. Total Environ..

[34] Fraser A., Macdonald-Wallis C., Tilling K., Boyd A., Golding J., Davey Smith G., Henderson J., Macleod J., Molloy L., Ness A. (2013). Cohort profile: The Avon Longitudinal Study of Parents and Children: ALSPAC mothers cohort. Int. J. Epidemiol..

[35] Boyd A., Golding J., Macleod J., Lawlor D.A., Fraser A., Henderson J., Molloy L., Ness A., Ring S., Davey Smith G. (2013). Cohort profile: The ’Children of the 90s’—The index offspring of the Avon Longitudinal Study of Parents and Children. Int. J. Epidemiol..

[36] University of Bristol Avon Longitudinal Study of Parents and Children. www.bris.ac.uk/alspac/.

[37] University of Bristol Avon Longitudinal Study of Parents and Children: Explore data and samples. www.bristol.ac.uk/alspac/researchers/our-data/.

[38] Rogers I., Emmett P. (1998). Diet during pregnancy in a population of pregnant women in South West England. ALSPAC Study Team. Avon Longitudinal Study of Pregnancy and Childhood. Eur. J. Clin. Nutr..

[39] Gregory J., Foster K., Tyler H., Wiseman M. (1990). The Dietary and Nutritional Survey of British Adults.

[40] Williams C., Birch E.E., Emmett P.M., Northstone K., Avon Longitudinal Study of Pregnancy and Childhood Study Team (2001). Stereoacuity at age 3.5 y in children born full-term is associated with prenatal and postnatal dietary factors: A report from a population-based cohort study. Am. J. Clin. Nutr..

[41] Northstone K., Emmett P., Rogers I. (2008). Dietary patterns in pregnancy and associations with socio-demographic and lifestyle factors. Eur. J. Clin. Nutr..

[42] Taylor C.M., Golding J., Hibbeln J., Emond A.M. (2013). Enviromental factors in relation to blood lead levels in pregnant women in the UK: The ALSPAC study. PLoS ONE.

[43] Centers for Disease Control and Prevention (2005). Third National Report on Human Exposure to Environmental Chemicals.

[44] Hornung R., Reed L.D. (1990). Estimation of average concentration in the prescence of nondetectable values. Appl. Occup. Environ. Hyg..

[45] Townsend P., Phillimore P., Beattie A. (1988). Health and Deprivation: Inequality and the North.

[46] Brion M.J., Ness A.R., Rogers I., Emmett P., Cribb V., Davey Smith G., Lawlor D.A. (2010). Maternal macronutrient and energy intakes in pregnancy and offspring intake at 10 y: Exploring parental comparisons and prenatal effects. Am. J. Clin. Nutr..

[47] Adams S.V., Newcomb P.A. (2014). Cadmium blood and urine concentrations as measures of exposure: NHANES 1999–2010. J. Expo. Sci. Environ. Epidemiol..

[48] Roels H., Hubermont G., Buchet J.P., Lauwerys R. (1978). Placental transfer of lead, mercury, cadmium, and carbon monoxide in women. III. Factors influencing the accumulation of heavy metals in the placenta and the relationship between metal concentration in the placenta and in maternal and cord blood. Environ. Res..

[49] Lauwerys R.R., Bernard A.M., Roels H.A., Buchet J.P. (1994). Cadmium: Exposure markers as predictors of nephrotoxic effects. Clin. Chem..

[50] Sanders A.P., Flood K., Chiang S., Herring A.H., Wolf L., Fry R.C. (2012). Towards prenatal biomonitoring in North Carolina: Assessing arsenic, cadmium, mercury, and lead levels in pregnant women. PLoS ONE.

[51] Gerhardsson L., Lundh T. (2010). Metal concentrations in blood and hair in pregnant females in southern Sweden. J. Environ. Health.

[52] Ebert-McNeill A., Clark S., Miller J., Birdsall P., Chandar M., Wu L., Cerny E., Hall P., Johnson M., Isales C. (2012). Cadmium intake and systemic exposure in postmenopausal women and age-matched men who smoke cigarettes. Toxicol. Sci. Off. J. Soc. Toxicol..

[53] Menai M., Heude B., Slama R., Forhan A., Sahuquillo J., Charles M.A., Yazbeck C. (2012). Association between maternal blood cadmium during pregnancy and birth weight and the risk of fetal growth restriction: The EDEN mother–child cohort study. Reprod. Toxicol..

[54] Schulz C., Angerer J., Ewers U., Kolossa-Gehring M. (2007). The German Human Biomonitoring Commission. Int. J. Hyg. Environ. Health.

[55] Ferrari P., Arcella D., Heraud F., Cappe S., Fabiansson S. (2013). Impact of refining the assessment of dietary exposure to cadmium in the European adult population. Food Addit. Contam. Part A Chem. Anal. Control Expo. Risk Assess..

[56] Food Standards Agency (2015). Total Diet Study of Metals and Other Elements in Foods.

[57] Kim K., Melough M.M., Vance T.M., Noh H., Koo S.I., Chun O.K. (2018). Dietary cadmium intake and sources in the US. Nutrients.

[58] Filippini T., Cilloni S., Malavolti M., Violi F., Malagoli C., Tesauro M., Bottecchi I., Ferrari A., Vescovi L., Vinceti M. (2018). Dietary intake of cadmium, chromium, copper, manganese, selenium and zinc in a Northern Italy community. J. Trace Elem. Med. Biol..

[59] Yu G., Zheng W., Wang W., Dai F., Zhang Z., Yuan Y., Wang Q. (2017). Health risk assessment of Chinese consumers to cadmium via dietary intake. J. Trace Elem. Med. Biol..

[60] Caspersen I.H., Thomsen C., Haug L.S., Knutsen H.K., Brantsaeter A.L., Papadopoulou E., Erlund I., Lundh T., Alexander J., Meltzer H.M. (2019). Patterns and dietary determinants of essential and toxic elements in blood measured in mid-pregnancy: The Norwegian Environmental Biobank. Sci. Total Environ..

[61] Krajcovicova-Kudladkova M., Ursinyova M., Masanova V., Bederova A., Valachovicova M. (2006). Cadmium blood concentrations in relation to nutrition. Cent. Eur. J. Public Health.

[62] Shawki A., Mackenzie B. (2010). Interaction of calcium with the human divalent metal-ion transporter-1. Biochem. Biophys. Res. Commun..

[63] Dix-Cooper L., Kosatsky T. (2018). Blood mercury, lead and cadmium levels and determinants of exposure among newcomer South and East Asian women of reproductive age living in Vancouver, Canada. Sci. Total Environ..

[64] Arbuckle T.E., Liang C.L., Morisset A.S., Fisher M., Weiler H., Cirtiu C.M., Legrand M., Davis K., Ettinger A.S., Fraser W.D. (2016). Maternal and fetal exposure to cadmium, lead, manganese and mercury: The MIREC study. Chemosphere.

[65] Newby P.K., Tucker K.L. (2004). Empirically derived eating patterns using factor or cluster analysis: A review. Nutr. Rev..

[66] Bocca B., Ruggieri F., Pino A., Rovira J., Calamandrei G., Martinez M.A., Domingo J.L., Alimonti A., Schuhmacher M. (2019). Human biomonitoring to evaluate exposure to toxic and essential trace elements during pregnancy. Part A. concentrations in maternal blood, urine and cord blood. Environ. Res..

[67] McGowan C.A., McAuliffe F.M. (2013). Maternal dietary patterns and associated nutrient intakes during each trimester of pregnancy. Public Health Nutr..

[68] Savard C., Lemieux S., Weisnagel S.J., Fontaine-Bisson B., Gagnon C., Robitaille J., Morisset A.S. (2018). Trimester-specific dietary intakes in a sample of French–Canadian pregnant women in comparison with national nutritional guidelines. Nutrients.

[69] Kopp-Hoolihan L.E., van Loan M.D., Wong W.W., King J.C. (1999). Longitudinal assessment of energy balance in well-nourished, pregnant women. Am. J. Clin. Nutr..

[70] Kubota K., Itoh H., Tasaka M., Naito H., Fukuoka Y., Muramatsu Kato K., Kohmura Y.K., Sugihara K., Kanayama N., Hamamatsu Birth Cohort Study Team (2013). Changes of maternal dietary intake, bodyweight and fetal growth throughout pregnancy in pregnant Japanese women. J. Obstet. Gynaecol. Res..

[71] Northstone K., Emmett P.M. (2008). A comparison of methods to assess changes in dietary patterns from pregnancy to 4 years post-partum obtained using principal components analysis. Br. J. Nutr..

[72] Suh S.Y., Lee J.H., Park S.S., Seo A.R., Ahn H.Y., Bae W.K., Lee Y.J., Yim E. (2013). Less healthy dietary pattern is associated with smoking in Korean men according to nationally representative data. J. Korean Med. Sci..

