# Supplementary material for: Maternal Diet During Pregnancy and Blood Cadmium Concentrations in an Observational Cohort of British Women
Source: Nutrients. 2020 Mar 26;12(4):904. doi: 10.3390/nu12040904 (PMC7230298; doi:10.3390/nu12040904)
Supplement: Supplementary file 1 [file nutrients-12-00904-s001.pdf]

Table S1 Food groups and individual foods combined for the analyses

Food group	Individual foods in group
Meats	Poultry, red meat, offal,
Fish	White fish, oily fish. Shellfish
Pulses	Pulses, bakes beans
Nuts	Nuts, tahini
Soyabean products	Bean curd, soya/SIM/non-meat
Root vegetables	Carrots, boiled/baked/roasted potatoes, other root vegetables
Leafy greens and green vegetables	Peas, leafy greens, other green vegetables, salad
Breads and cereals	Oat cereal, bran cereal, other cereal, crispbread
Cakes and biscuits	Cakes, buns, biscuits
Pasta and rice	Pasta, rice
Pies/pastries	Pizza, pies, pastries

Table S2 Sensitivity analysis 1: Blood cadmium concentrations in pregnant women enrolled in ALSPAC by indicators of socio-economic positioning and lifestyle (complete case analysis excluding smokers in the first trimester, n=1687)

Variable	Observations n (%)	n (%)		Odds ratio (95% CI)	*p* value
		B-Cd <median	B-Cd ≥ median		
**Maternal age (years)**					
≤19	25 (1.48)	13 (1.09)	12 (2.40)	1.00	
20 to <25	169 (11.62)	122 (10.27)	74 (14.83)	0.66 (0.28, 1.52)	
25 to <30	656 (38.89)	471 (39.65)	185 (37.07)	0.43 (0.19, 0.95)	
30 to <35	605 (35.86)	439 (36.95)	166 (33.27)	0.41 (0.18, 0.92)	
≥35	205 (12.15)	143 (12.04)	62 (12.42)	0.47 (0.20, 1.09)	0.019
**Maternal education**					
None/CSE	340 (20.15)	223 (18.77)	117 (23.45)	1.00	
Vocational/O-level	1011 (59.93)	705 (59.34)	306 (61.32)	0.83 (0.64, 1.07)	
A-level and above	336 (19.92)	260 (21.89)	76 (15.23)	0.56 (0.40, 0.78)	0.002
**Townsend score**					
1	568 (33.67)	414 (34.85)	154 (30.86)	1.00	
2	278 (16.48)	204 (17.17)	74 (14.83)	0.98 (0.71, 1.35)	
3	441 (26.14)	308 (26.93)	133 (26.65)	1.16 (0.88, 1.53)	
4	400 (23.71)	262 (22.05)	138 (27.66)	1.42 (1.07, 1.87)	0.058
**Alcohol consumption**					
No	786 (46.59)	545 (45.88)	241 (48.30)	1.00	
Yes	901 (53.41)	643 (54.12)	258 (51.70)	0.91 (0.74, 1.12)	0.363
**BMI**					
Normal/underweight	1341 (79.49)	950 (79.97)	391 (78.36)	1.00	
Overweight	261 (15.47)	183 (15.40)	78 (15.63)	1.04 (0.77, 1.38)	
Obese	85 (5.04)	55 (4.63)	30 (6.01)	1.33 (0.84, 2.10)	0.493
**Vegetarian diet**					
Never	1408 (85.39)	1008 (86.75)	400 (85.39)	1.00	
In the past	130 (7.88)	84 (7.23)	46 (7.88)	1.38 (0.95, 2.01)	
Present	111 (6.73)	70 (6.02)	41 (6.73)	1.48 (0.99, 2.21)	0.057

Educational attainment: none/Certificate of School Education, vocational/Ordinary level, Advanced level and above.
Townsend score is a measure of deprivation: 1 is the least deprived, score 4 the most deprived [45].
Social class: I, higher managerial, administrative or professional; II, intermediate managerial, administrative or professional; III non-manual, supervisory or clerical and junior management, administrative or professional; III manual, skilled manual workers; IV, semi-skilled and unskilled manual workers; V, casual or lowest grade workers.
Median B-Cd 0.23 µg/l.
BMI (body mass index): normal/underweight <24.9; overweight 25.0–29.9; obese ≥30.0 kg/m^2.

Table S3 Sensitivity analysis 1: Associations of dietary patterns with blood cadmium concentrations in pregnant women enrolled in ALSPAC (complete case analysis excluding those who smoked in the first trimester, n=1687)

Pattern	Quartile	Median B-Cd (μg/l)	n (%)		OR (95% CI)	
			B-Cd < median	B-Cd ≥ median	Unadjusted Model 1	Adjusted Model 2
		Overall median 0.23				
Health conscious	1	0.25	171 (14.4)	101 (20.2)	1.00	1.00
	2	0.24	252 (21.2)	134 (26.9)	0.90 (0.65-1.24)	0.98 (0.70-1.37)
	3	0.22	357 (30.1)	140 (28.1)	0.66 (0.48-0.91)	0.74 (0.53-1.05)
	4	0.21	408 (34.3)	124 (24.9)	0.51 (0.37-0.71)	0.60 (0.41-0.87)
					P trend <0.001	P trend=0.001
Traditional	1	0.23	275 (23.2)	129 (25.9)	1.00	1.00
	2	0.22	297 (25.0)	109 (21.8)	0.78 (0.58-1.06)	0.81 (0.60-1.11)
	3	0.23	308 (25.9)	126 (25.3)	0.87 (0.65-1.17)	0.88 (0.66-1.20)
	4	0.22	308 (25.9)	135 (27.1)	0.93 (0.70-1.25)	0.96 (0.71-1.30)
					P trend=0.851	P trend=0.995
Processed	1	0.22	357 (30.1)	132 (26.5)	1.00	1.00
	2	0.23	336 (28.3)	127 (25.5)	1.02 (0.77-1.36)	1.02 (0.76-1.37)
	3	0.24	261 (22.0)	140 (28.1)	1.45 (1.09-1.93)	1.45 (1.07-1.96)
	4	0.22	234 (19.7)	100 (20.0)	1.16 (0.85-1.57)	1.18 (0.82-1.68)
					P trend=0.076	P trend=0.094
Confectionery	1	0.23	289 (24.3)	122 (24.5)	1.00	1.00
	2	0.23	308 (25.9)	132 (26.5)	1.02 (0.76-1.36)	1.08 (0.80-1.46)
	3	0.22	313 (26.4)	130 (26.1)	0.98 (0.73-1.32)	1.10 (0.81-1.51)
	4	0.23	278 (23.4)	115 (23.1)	0.98 (0.72-1.33)	1.09 (0.76-1.56)
					P trend=0.847	P trend=0.703
Vegetarian	1	0.22	359 (30.2)	130 (26.1)	1.00	1.00
	2	0.23	299 (25.2)	128 (25.7)	1.18 (0.89-1.58)	1.12 (0.83-1.51)
	3	0.22	252 (21.2)	110 (22.0)	1.21 (0.89-1.63)	1.12 (0.82-1.54)
	4	0.23	278 (23.4)	131 26.3)	1.30 (0.97-1.74)	1.25 (0.93-1.69)
					P trend=0.079	P trend=0.124

Model 2 adjusted for maternal age, maternal education, Townsend score, BMI, energy intake, alcohol consumption and haemoglobin levels.

Table S4 Sensitivity analysis 1: Associations of frequency of intakes of foods and food group with blood cadmium concentrations in pregnant women enrolled in ALSPAC (complete case analysis excluding those who smoked in the first trimester, n=1687)

	n (%)		OR (95% CI)	
	B-Cd < median	B-Cd ≥ median	Unadjusted Model 1	Adjusted Model 2
**Total n**	1188	499		
**All meats combined**				
≤ Once in 2 weeks	146 (12.3)	83 (16.6)	1.00	1.00
≤3 times per week	741 (62.4)	311 (62.3)	0.74 (0.55-1.00)	0.76 (0.56-1.03)
≥4 times per week for at least one group	301 (25.3)	105 (21.0)	0.61 (0.43-0.87)	0.67 (0.46-0.97)
			P trend = 0.008	P trend = 0.031
**All fish**				
≤ Once in 2 weeks	535.1188 (45.0)	264 (52.9)	1.00	1.00
≥1 to 3 times per week	603 (50.8)	217 (43.5)	0.73 (0.59-0.9)	0.76 (0.61-0.96)
≥4 to 7 times per week	50 (4.2)	18 (3.6)	0.73 (0.42-1.28)	0.76 (0.43-1.36)
			P trend = 0.006	P trend = 0.024
**Milk (glasses per day)[b]**				
None/rarely	501 (43.4)	231 (47.8)	1.00	1.00
1 to 2 glasses per day	566 (49.0)	212 (43.9)	0.81 (0.65-1.01)	0.77 (0.62-0.98)
≥3 glasses per day	88 (7.6)	40 (8.3)	0.99 (0.66-1.48)	0.83 (0.55-1.27)
			P trend = 0.263	P trend = 0.074
**All pulses combined**				
≤ Once in 2 weeks	135 (11.36)	77 (15.4)	1.00	1.00
≤3 times per week	942 (79.29)	381 (76.4)	0.71 (0.52-0.96)	0.74 (0.54-1.01)
≥4 times per week for at least one group	111 (9.34)	41 (8.2)	0.65 (0.41-1.02)	0.68 (0.43-1.10)
			P trend = 0.036	P trend = 0.089
**All nuts combined**				
Never/rarely	731 (61.5)	326 (65.3)	1.00	1.00
≤ Once in 2 weeks	329 (27.7)	115 (23.1)	0.78 (0.61-1.00)	0.84 (0.65-1.09)
≥1 to 3 times per week	128 (10.8)	58 (11.6)	1.02 (0.73-1.42)	1.12 (0.79-1.60)
			P trend = 0.042	P trend = 0.981
**Soybean products**				
Never or rarely	1075 (90.5)	440 (88.2)	1.00	1.00
≤ Once in 2 weeks	113 (9.5)	59 (11.8)	1.28 (0.91-1.78)	1.33 (0.94-1.88)
			P trend = 0.153	P trend = 0.081
**Root vegetables**				
Never or rarely	34 (2.9)	22 (4.4)	1.00	1.00

	n (%)		OR (95% CI)	
	B-Cd < median	B-Cd ≥ median	Unadjusted Model 1	Adjusted Model 2
≤ One to 3 times per week per food	678 (57.8)	281 (56.3)	0.63 (0.36-1.10)	0.70 (0.40-1.24)
≥4 to 7 times per week	467 (39.3)	196 (39.3)	0.65 (0.37-1.14)	0.77 (0.43-1.38)
			P trend = 0.587	P trend = 0.862
**All leafy green and green vegetables**				
≤1 to 3 times per week	233 (19.6)	129 (25.9)	1.00	1.00
≥4 times per week	955 (80.4)	370 (74.2)	0.70 (0.55-0.90)	0.74 (0.57-0.95)
			P trend = 0.002	P trend = 0.008
**Combined breads and cereals**				
≤ Once a week	99 (8.3)	59 (11.8)	1.00	1.00
≤One to 3 times per week per food	316 (26.6)	135 (27.1)	0.72 (0.49-1.05)	0.78 (0.53-1.14)
≥4 to 7 times per week	773 (65.1)	305 (61.1)	0.66 (0.47-0.94)	0.74 (0.52-1.07)
			P trend = 0.035	P trend = 0.136
**All cakes and biscuits**				
≤ Once a week	180 (15.2)	98 (19.6)	1.00	1.00
≤ One to 3 times per week per food	592 (49.8)	236 (47.3)	0.73 (0.55-0.98)	0.78 (0.58-1.06)
≥4 to 7 times per week	416 (35.0)	165 (33.1)	0.73 (0.54-0.99)	0.82 (0.58-1.15)
			P trend = 0.081	P trend = 0.298
**All pies and pastries**				
Never or rarely	240 (20.2)	107 (21.4)	1.00	1.00
≤ Once in 2 weeks	679 (57.2)	288 (57.7)	0.95 (0.73-1.24)	0.97 (0.74-1.28)
≥1 to 3 times per week	269 (22.6)	104 (20.8)	0.87 (0.63-1.20)	0.84 (0.59-1.18)
			P trend = 0.383	P trend = 0.268
**All pasta and rice**				
Never or rarely	88 (7.4)	50 (10.0)	1.00	1.00
≤ Once in 2 weeks	329 (27.7)	151 (30.3)	0.81 (0.54-1.20)	0.87 (0.58-1.30)
≥1 to 3 times per week	771 (64.9)	298 (59.7)	0.68 (0.47-0.99)	0.83 (0.56-1.23)
			P trend = 0.023	P trend = 0.278

Model 2 adjusted for maternal age, maternal education, Townsend score, BMI, energy intake, alcohol consumption and haemoglobin levels.

[a]A standard glass of milk is 200 mL.

Median B-Cd 0.23 µg/l.

Calcium intake (quartiles): p for trend 0.806 in adjusted model (data not shown).

Table S5 Sensitivity analysis 2: Blood cadmium concentrations in pregnant women enrolled in ALSPAC by indicators of socio-economic positioning and lifestyle (complete case analysis excluding those who smoked in the first trimester and pre-pregnancy, n=1518)

Variable	Observations n (%)	n (%)		Odds ratio (95% CI)	*p* value
		B-Cd <median	B-Cd ≥ median		
**Maternal age (years)**					
≤19	16 (1.1)	10 (1.2)	6 (0.9)	1.00	0.095
20 to <25	157 (10.3)	83 (10.0)	74 (10.7)	1.49 (0.52, 4.29)	
25 to <30	601 (39.6)	343 (41.4)	258 (37.4)	1.25 (0.45, 3.49)	
30 to <35	550 (36.2)	301 (36.4)	249 (36.1)	1.38 (0.49, 3.85)	
≥35	194 (12.8)	91 (11.0)	103 (14.9)	1.89 (0.66, 5.39)	
**Maternal education**					
None/CSE	298 (19.6)	163 (19.7)	135 (19.6)	1.00	0.222
Vocational/O-level	911 (60.1)	481 (58.1)	430 (62.3)	1.08 (0.83, 1.40)	
A-level and above	309 (20.4)	184 (22.2)	125 (18.1)	0.82 (0.59, 1.13)	
**Townsend score**					
1	527 (34.7)	308 (37.2)	219 (31.7)	1.00	0.079
2	255 (16.8)	125 (15.1)	130 (18.8)	1.46 (1.08, 1.98)	
3	395 (26.0)	222 (26.8)	173 (25.1)	1.10 (0.84, 1.42)	
4	341 (22.5)	173 (20.9)	168 (24.3)	1.37 (1.04, 1.80)	
**Alcohol consumption**					
No	711 (46.8)	379 (45.8)	332 (48.1)	1.00	0.362
Yes	807 (53.2)	449 (54.2)	358 (51.9)	0.91 (0.74, 1.11)	
**BMI**					
Normal/underweight	1209 (79.6)	664 (80.2)	545 (79.0)	1.00	0.414
Overweight	232 (15.3)	126 (15.2)	106 (15.4)	1.03 (0.77, 1.36)	
Obese	77 (5.1)	38 (4.6)	39 (5.7)	1.25 (0.79, 1.98)	
**Vegetarian diet**					
Never	1282 (84.5)	709 (87.6)	573 (84.9)	1.00	0.123
In the past	109 (7.2)	55 (6.8)	54 (8.0)	1.22 (0.82, 1.80)	
Present	93 (6.1)	45 (5.6)	48 (7.1)	1.32 (0.87, 2.01)	

Educational attainment: none/Certificate of School Education, vocational/Ordinary level, Advanced level and above.

Townsend score is a measure of deprivation: 1 is the least deprived, score 4 the most deprived [45].

Social class: I, higher managerial, administrative or professional; II, intermediate managerial, administrative or professional; III non-manual, supervisory or clerical and junior management, administrative or professional; III manual, skilled manual workers; IV, semi-skilled and unskilled manual workers; V, casual or lowest grade workers.

Median B-Cd 0.22 µg/l.

BMI (body mass index): normal/underweight <24.9; overweight 25.0–29.9; obese ≥30.0 kg/m^2.

Table S6 Sensitivity analysis 2: Associations of dietary patterns with blood cadmium concentrations in pregnant women enrolled in ALSPAC (complete case analysis excluding current smokers and pre-pregnancy smokers, n=1518)

Pattern	Quartile	Median B-Cd (µg/l)	n (%)		OR (95% CI)	
			B-Cd < median	B-Cd ≥ median	Unadjusted Model 1	Adjusted Model 2
		Overall median 0.22				
Health conscious	1	0.23	115 (13.9)	119 (17.2)	1.00	1.00
	2	0.22	179 (21.6)	161 (23.3)	0.87 (0.62, 1.21)	0.85 (0.60, 1.19
	3	0.22	244 (29.5)	210 (30.4)	0.83 (0.61, 1.14)	0.79 (0.56, 1.11
	4	0.20	290 (35.0)	200 (29.0)	0.67 (0.49, 0.91)	0.61 (0.41, 0.88)
					P trend = 0.008	P trend = 0.006
Traditional	1	0.22	188 (22.7)	167 (24.2)	1.00	1.00
	2	0.21	207 (25.0)	162 (23.5)	0.88 (0.66, 1.18)	0.87 (0.64, 1.16)
	3	0.22	208 (24.1)	182 (26.4)	0.99 (0.74, 1.31)	0.97 (0.73, 1.30)
	4	0.21	225 (27.2)	179 (25.9)	0.90 (0.67, 1.19)	0.88 (0.66, 1.18)
					P trend = 0.979	P trend =0.575
Processed	1	0.21	252 (30.4)	196 (28.4)	1.00	1.00
	2	0.22	227 (27.4)	190 (27.5)	1.08 (0.82, 1.41)	1.10 (0.84, 1.45)
	3	0.22	178 (21.5)	174 (25.2)	1.26 (0.95, 1.66)	1.27 (0.95, 1.71)
	4	0.21	171 (20.7)	130 (18.8)	0.98 (0.73, 1.31)	1.03 (0.74, 1.45)
					P trend =0.708	P trend = 0.503
Confectionery	1	0.22	197 (23.8)	172 (24.9)	1.00	1.00
	2	0.22	214 (25.8)	176 (25.5)	0.94 (0.71, 1.25)	0.98 (0.73, 1.31)
	3	0.21	229 (27.7)	175 (25.4)	0.88 (0.66, 1.16)	0.91 (0.68, 1.23)
	4	0.22	188 (22.7)	167 (24.2)	1.02 (0.76, 1.36)	1.08 (0.77, 1.52)
					P trend =0.939	P trend =0.828
Vegetarian	1	0.21	248 (30.0)	213 (30.9	1.00	1.00
	2	0.22	211 (25.5)	171 (24.8)	0.94 (0.72, 1.24)	0.91 (0.69, 1.21)
	3	0.21	182 (22.0)	136 (19.7)	0.87 (0.65, 1.17)	0.85 (0.63, 1.14)
	4	0.22	187 (22.6)	170 (24.6)	1.06 (0.80, 1.40)	1.03 (0.78, 1.37)
					P trend=0.996	P Trend = 0.998

Model 2 adjusted for maternal age, maternal education, Townsend score, BMI, energy intake, alcohol consumption and smoking status during first trimester, and haemoglobin levels.

Table S7 Sensitivity analysis 2: Associations of frequency of intakes of foods and food group with blood cadmium concentrations in pregnant women enrolled in ALSPAC (complete case analysis excluding those who smoked in the first trimester and pre-pregnancy, n=1518)

	n (%)		OR (95% CI)	
	**B-Cd < median**	**B-Cd ≥ median**	**Unadjusted Model 1**	**Adjusted Model 2**
**Total n**	828 (54.4)	690 (45.5)		
**All meats combined**				
≤ Once in 2 weeks	93 (11.2)	100 (14.5)	1.00	1.00
≤3 times per week	523 (63.2)	424 (61.4)	0.75 (055, 1.03)	0.77 (0.56, 1.05)
≥4 times per week for at least one group	212 (25.6)	166 (24.4)	0.73 (0.51, 1.03)	0.76 (0.52, 1.09)
			P trend = 0.199	P trend =0.199
**All fish**				
≤ Once in 2 weeks	359 (43.4)	355 (51.4)	1.00	1.00
≥1 to 3 times per week	429 (51.8)	315 (45.7)	0.74 (0.60, 0.91)	0.74 (0.60, 0.91)
≥4 to 7 times per week	40 (4.8)	20 (2.9)	0.50 (0.29, 0.88)	0.52 (0.29, 0.91)
			P trend =0.001	P trend =0.001
**Milk (glasses per day)[b]**				
None/rarely	349 (43.4)	312 (46.4)	1.00	1.00
1 to 2 glasses per day	399 (49.6)	303 (45.0)	0.85 (0.69, 1.05)	0.86 (0.69, 1.06)
≥3 glasses per day	56 (7.0)	58 (8.6)	1.16 (0.78, 1.72)	1.13 (0.75, 1.70)
			P trend =0.162	P trend =0.221
**All pulses combined**				
≤ Once in 2 weeks	92 (11.1)	96 (13.9)	1.00	1.00
≤3 times per week	660 (79.7)	537 (77.8)	0.78 (0.57, 1.06)	0.79 (0.58, 1.08)
≥4 times per week for at least one group	76 (9.2)	57 (8.3)	0.72 (0.46, 1.12)	0.74 (0.47, 1.16)
			P trend =0.234	P trend =0.296
**All nuts combined**				
Never/rarely	525 (63.4)	423 (61.3)	1.00	1.00
≤ Once in 2 weeks	215 (26.0)	187 (27.1)	1.08 (0.85, 1.36)	1.09 (0.86, 1.39)
≥1 to 3 times per week	88 (10.6)	80 (11.6)	1.13 (0.81, 1.57)	1.14 (0.81, 1.61)
			P trend =0.682	P trend =0.634
**Soybean products**				
Never or rarely	755 (91.2)	617 (89.4)	1.00	1.00
≤ Once in 2 weeks	91.2 (8.8)	89.4 (10.6)	1.22 (0.87, 1.72)	0.22 (0.86, 1.73)
			P trend = 0.247	P trend = 0.257
**Root vegetables**				
Never or rarely	23 (2.8)	22 (3.2)	1.00	1.00

	n (%)		OR (95% CI)	
	B-Cd < median	B-Cd ≥ median	Unadjusted Model 1	Adjusted Model 2
≤ One to 3 times per week per food	469 (56.6)	398 (57.7)	0.89 (0.49, 1.62)	0.86 (0.47, 1.58)
≥4 to 7 times per week	336 (40.6)	270 (39.1)	0.84 (0.46, 1.54)	0.83 (0.44, 1.54)
			P trend =0.786	P trend =0.816
**All leafy green and green vegetables**				
≤1 to 3 times per week	164 (19.8)	145 (21.0)	1.00	1.00
≥4 times per week	664 (80.2)	545 (79.0)	0.93 (0.72, 1.19)	0.91 (0.70, 1.18)
			P trend = 0.561	P trend = 0.473
**Combined breads and cereals**				
≤ Once a week	59 (7.1)	74 (10.7)	1.00	1.00
≤One to 3 times per week per food	230 (27.8)	178 (25.8)	0.62 (0.42, 0.92)	0.64 (0.43, 0.96
≥4 to 7 times per week	239 (65.1)	438 (63.5)	0.65 (0.45, 0.93)	0.68 (0.47, 0.99)
			P trend = 0.045	P trend = 0.086
**All cakes and biscuits**				
≤ Once a week	126 (15.2)	115 (16.7)	1.00	1.00
≤ One to 3 times per week per food	420 (50.7)	330 (47.8)	0.86 (0.64, 1.15)	0.88 (0.66, 1.19)
≥4 to 7 times per week	282 (34.1)	245 (35.5)	0.95 (0.70, 1.29)	1.00 (0.71, 1.39)
			P trend = 0.505	P trend = 0.517
**All pies and pastries**				
Never or rarely	168 (20.3)	145 (21.0)	1.00	1.00
≤ Once in 2 weeks	472 (57.0)	395 (57.2)	0.97 (0.75, 1.26)	1.00 (0.77, 1.30)
≥1 to 3 times per week	188 (22.7)	150 (21.7)	0.92 (0.68, 1.26)	0.93 (0.68, 1.30)
			P trend = 0.879	P trend = 0.896
**All pasta and rice**				
Never or rarely	63 (7.6)	61 (8.8)	1.00	1.00
≤ Once in 2 weeks	227 (27.4)	191 (27.7)	0.87 (0.58, 1.30)	0.91 (0.61, 1.37)
≥1 to 3 times per week	538 (65.0)	438 (63.5)	0.84 (0.58, 1.22)	0.88 (0.60, 1.31)
			P trend = 0.657	P trend = 0.820

Model 2 adjusted for maternal age, maternal education, Townsend score, BMI, energy intake, alcohol consumption and haemoglobin levels.

[a]A standard glass of milk is 200 mL.

Median B-Cd 0.22 µg/l.

Calcium intake (quartiles): p for trend 0.334 in adjusted model (data not shown).

Table S8 Sensitivity analysis 3: Blood cadmium concentrations in pregnant women enrolled in ALSPAC by indicators of socio-economic positioning and lifestyle (complete case analysis excluding <LOD, n=1505)

Variable	Observations n (%)	n (%)		Odds ratio (95% CI)	*p* value
		B-Cd < median	B-Cd ≥ median		
**Maternal age (years)**					
≤19	46 (3.1)	11 (1.5)	35 (4.6)	1.00	<0.001
20 to <25	251 (16.7)	91 (12.2)	160 (21.1)	0.55 (0.27-1.14)	
25 to <30	574 (38.1)	284 (38.0)	290 (38.3)	0.32 (0.16-0.64)	
30 to <35	465 (30.9)	264 (35.3)	201 (26.6)	0.24 (0.12-0.48)	
≥35	169 (11.2)	98 (13.1)	71 (9.4)	0.23 (0.11-0.48)	
**Maternal education**					
None/CSE	416 (27.6)	154 (20.6)	262 (34.6)	1.00	<0.001
Vocational/O-level	881 (58.5)	456 (61.0)	425 (56.1)	0.55 (0.43-0.67)	
A-level and above	208 (13.8)	138 (18.4)	70 (9.2)	0.30 (0.21-0.42)	
**Townsend score**					
1	392 (26.0)	218 (29.1)	174 (23.0)	1.00	<0.001
2	248 (16.5)	144 (19.3)	104 (13.7)	0.91 (0.66-1.25)	
3	402 (26.7)	198 (26.5)	204 (26.9)	1.39 (0.98-1.71)	
4	463 (30.8)	188 (25.1)	275 (36.3)	1.83 (1.40-2.41)	
**Smoking status in 1st trimester**					
No	1031 (68.5)	416 (97.9)	615 (56.9)	1.00	<0.001
Yes	474 (31.5)	9 (2.1)	464 (43.0)	18.41 (12.24, 27.68)	
**Smoked regularly pre-pregnancy**					
No	873 (58.0)	402 (94.6)	471 (43.6)	1.00	<0.001
Yes	632 (42.0)	23 (5.4)	609 (56.4)	34.35 (17.55, 67.21)	
**Alcohol consumption**					
No	131 (68.5)	335 (44.8)	319 (42.1)	1.00	0.300
Yes	474 (31.5)	413 (55.2)	438 (57.9)	1.11 (0.91-1.37)	
**BMI**					
Normal/underweight	1168 (77.6)	585 (78.2)	583 (77.0)	1.00	0.161
Overweight	256 (17.2)	131 (17.5)	125 (16.5)	0.96 (0.73-1.26)	
Obese	81 (5.4)	32 (4.3)	49 (6.5)	1.54 (0.97-2.43)	
**Vegetarian diet**					
Never	1246 (82.8)	620 (84.8)	620 (84.1)	1.00	0.461
In the past	135 (9.0)	61 (8.3)	74 (9.9)	1.20 (0.84-0.72)	
Present	94 (6.2)	50 (6.8)	44 (5.9)	0.87 (0.57-1.33)	

Educational attainment: none/Certificate of School Education, vocational/Ordinary level, Advanced level and above.

Townsend score is a measure of deprivation: 1 is the least deprived, score 4 the most deprived [45].

Social class: I, higher managerial, administrative or professional; II, intermediate managerial, administrative or professional; III non-manual, supervisory or clerical and junior management, administrative or professional; III

manual, skilled manual workers; IV, semi-skilled and unskilled manual workers; V, casual or lowest grade workers.

Median B-Cd 0.37 µg/l.

BMI (body mass index): normal/underweight <24.9; overweight 25.0–29.9; obese ≥30.0 kg/m^2.

Table S9 Sensitivity analysis 3: Associations of dietary patterns with blood cadmium concentrations in pregnant women enrolled in ALSPAC (complete case analysis excluding <LOD, n=1505)

**Pattern**	**Quartile**	**Median B-Cd (µg/l)**	**n (%)**		**OR (95% CI)**	
			**B-Cd < median**	**B-Cd ≥ median**	**Unadjusted Model 1**	**Adjusted Model 2**
		Overall median 0.37				
Health conscious	1	0.75	122 (16.3)	254 (33.6)	1.00	1.00
	2	0.38	180 (24.1)	196 (25.9)	0.52 (0.39-0.70)	0.79 (0.53-1.16)
	3	0.33	215 (28.7)	162 (21.4)	0.36 (0.27-0.49)	0.71 (0.48-1.07)
	4	0.30	231 (30.9)	145 (19.2)	0.30 (0.22-0.41)	0.75 (0.48-1.17)
					P trend <0.001	P trend = 0.203
Traditional	1	0.37	180 (24.1)	196 (25.9)	1.00	1.00
	2	0.35	197 (26.3)	179 (23.6)	0.83 (0.63-1.11)	0.84 (0.58-1.22)
	3	0.37	187 (25.0)	190 (25.1)	0.93 (0.70-1.24)	0.89 (0.62-1.29)
	4	0.37	184 (24.6)	192 (25.4)	0.96 (0.72-1.28)	1.11 (0.77-1.59)
					P trend = 0.972	P trend = 0.543
Processed	1	0.33	208 (27.8)	168 (22.2)	1.00	1.00
	2	0.34	201 (26.9)	175 (23.1)	1.08 (0.81-1.44)	0.85 (0.60-1.22)
	3	0.36	189 (25.3)	188 (24.8)	1.23 (0.93-1.64)	0.94 (0.65-1.36)
	4	0.50	150 (20.1)	226 (29.9)	1.87 (1.40-2.50)	0.91 (0.61-1.44)
					P trend <0.001	P trend = 0.831
Confectionery	1	0.43	160 (21.4)	216 (28.5)	1.00	1.00
	2	0.37	187 (25.0)	189 (25.0)	0.75 (0.56-1.00)	0.76 (0.53-1.10)
	3	0.33	205 (27.4)	172 (22.7)	0.62 (0.47-0.83)	0.75 (0.52-1.10)
	4	0.36	196 (26.2)	180 (23.8)	0.68 (0.51-0.91)	0.68 (0.45-1.05)
					P trend = 0.004	P trend = 0.090
Vegetarian	1	0.32	221 (29.5)	155 (20.5)	1.00	1.00
	2	0.36	194 (25.9)	182 (20.4)	1.34 (1.00-1.78)	1.14 (0.78-1.65)
	3	0.45	153 (20.5)	224 (29.6)	2.09 (1.56-2.79)	1.48 (1.02-2.17)
	4	0.38	180 (24.1)	196 (25.9)	1.55 (1.16-2.07)	1.40 (0.97-2.01)
					P trend <0.001	P trend = 0.034

Model 2 adjusted for maternal age, maternal education, Townsend score, BMI, energy intake, alcohol consumption and smoking status during first trimester, and haemoglobin levels.

Table S10 Blood Cd concentration in participants by vegetarian status in pregnant women enrolled in ALSPAC (in complete cases and in complete cases excluding those with values below the limit of detection)

	**Below median B-Cd**	**Above median B-Cd**	***p* value**
**Complete cases**			
Never	922 (86.5)	890 (84.2)	0.244
In the past	80 (7.5)	100 (9.5)	
Present	64 (6.0)	1057 (6.3)	
**Complete cases excluding those with B-Cd <LOD**			
Never	620 (84.8)	626 (84.1)	0.461
In the past	61 (8.3)	74 (9.9)	
Present	50 (6.8)	44 (5.9)	

Values are n (%).

Chi-squared test.

Median B-Cd 0.26 µg/l for complete cases; median B-Cd 0.37 µg/l for complete cases excluding those <LOD.

Table S11 Sensitivity analysis 3: Associations of frequency of intakes of foods and food group with blood cadmium concentrations in pregnant women enrolled in ALSPAC (complete case analysis excluding <LOD, n=1505)

	n (%)		OR (95% CI)	
	B-Cd < median	B-Cd ≥ median	Unadjusted Model 1	Adjusted Model 2
**Total n**	748	757		
**All meats combined**				
≤ Once in 2 weeks	104 (14.0)	121 (16.0)	1.00	1.00
≤3 times per week	468 (62.6)	487 (64.3)	0.89 (0.67-1.20	0.79 (0.55-1.13)
≥4 times per week for at least one group	176 (23.5)	149 (19.7)	0.72 (0.52-1.02)	0.69 (0.44-1.08)
			P trend = 0.056	P trend = 0.112
**All fish**				
≤ Once in 2 weeks	356 (47.6)	438 (57.9)	1.00	1.00
≥1 to 3 times per week	370 (49.5)	294 (38.8)	0.65 (0.53-0.80)	0.75 (0.57-0.98)
≥4 to 7 times per week	22 (2.9)	25 (3.3)	0.92 (0.51-0.67)	1.24 (0.80-1.06)
			P trend = 0.001	P trend = 0.169
**Milk (glasses per day)**[a]				
None/rarely	339 (46.7)	304 (41.6)	1.00	1.00
1 to 2 glasses per day	367 (46.4)	352 (48.2)	1.17 (0.94-1.44)	1.03 (0.78-1.36)
≥3 glasses per day	50 (6.9)	75 (10.3)	1.67 (1.13-2.47)	1.35 (0.82-2.23)
			P trend = 0.011	P trend = 0.375
**All pulses combined**				
≤ Once in 2 weeks	90 (12.0)	112 (14.8)	1.00	1.00
≤3 times per week	601 (80.3)	582 (76.9)	0.78 (0.58-1.05)	0.67 (0.46-0.97)
≥4 times per week for at least one group	57 (7.6)	63 (8.3)	0.89 (0.57-1.40)	1.08 (0.62-1.90)
			P trend = 0.384	P trend = 0.943
**All nuts combined**				
Never/rarely	452 (60.4)	518 (68.4)	1.00	1.00
≤ Once in 2 weeks	214 (28.6)	173 (22.9)	0.71 (0.56-0.89)	0.78 (0.57-1.06)
≥1 to 3 times per week	82 (11.0)	66 (8.7)	0.70 (0.50-0.99)	1.22 (0.78-1.04)
			P trend = 0.003	P trend = 0.970
**Soybean products**				
Never or rarely	671 (89.7)	687 (90.8)	1.00	1.00
≤ Once in 2 weeks	70 (10.3)	77 (9.2)	0.89 (0.63-1.5)	0.91 (0.79-1.05)
			P trend = 0.494	P trend = 0.083
**Root vegetables**				
Never or rarely	24 (3.2)	49 (6.5)	1.00	1.00

	n (%)		OR (95% CI)	
	**B-Cd < median**	**B-Cd ≥ median**	**Unadjusted Model 1**	**Adjusted Model 2**
≤ One to 3 times per week per food	435 (58.2)	476 (62.9)	0.54 (0.32-0.89)	1.44 (0.74-2.79)
≥4 to 7 times per week	289 (38.6)	232 (30.6)	0.39 (0.23-0.66)	0.98 (0.74. 1.29)
			P trend <0.001	P trend = 0.658
**All leafy green and green vegetables**				
≤1 to 3 times per week	167 (22.3)	215 (28.4)	1.00	1.00
≥4 times per week	581 (77.7)	542 (71.6)	0.73 (0.57-0.92)	0.91 (0.68-1.23)
			P trend = 0.007	P trend = 0.529
**Combined breads and cereals**				
≤ Once a week	79 (10.6)	138 (18.2)	1.00	1.00
≤One to 3 times per week per food	189 (25.3)	232 (30.6)	0.70 (0.50-0.98)	0.98 (0.63-1.53)
≥4 to 7 times per week	480 (64.2)	387 (51.1	0.46 (0.34-0.63)	0.85 (0.57-1.29)
			P trend <0.001	P trend = 0.330
**All cakes and biscuits**				
≤ Once a week	106 (14.2)	178 (23.5)	1.00	1.00
≤ One to 3 times per week per food	374 (50.0)	366 (48.3)	0.58 (0.44-0.77)	0.58 (0.40-0.82)
≥4 to 7 times per week	268 (35.8)	213 (28.1)	0.47 (0.35-0.64)	0.58 (0.38-0.88)
			P trend <0.001	P trend = 0.023
**All pies and pastries**				
Never or rarely	135 (18.0)	162 (21.4)	1.00	1.00
≤ Once in 2 weeks	431 (57.6)	394 (52.0)	0.76 (0.58-0.99)	0.74 (0.53-1.03)
≥1 to 3 times per week	182 (24.3)	201 (26.6)	0.92 (0.68-1.25)	0.55 (0.36-0.84)
			P trend = 0.743	P trend = 0.005
**All pasta and rice**				
Never or rarely	135 (18.0)	162 (21.4)	1.00	1.00
≤ Once in 2 weeks	431 (57.6)	394 (52.0)	0.56 (0.39-0.82)	0.61 (0.38-0.99)
≥1 to 3 times per week	182 (24.3)	201 (26.6)	0.42 (0.30-0.60)	0.66 (0.42-1.04)
			P trend <0.001	P trend = 0.251

Model 2 adjusted for maternal age, maternal education, Townsend score, BMI, energy intake, alcohol consumption and smoking status during first trimester, and haemoglobin levels.

[a]A standard glass of milk is 200 mL.

Calcium intake (quartiles): p for trend 0.375 in adjusted model (data not shown).
